# Photovoltaic Performance of FAPbI_3_ Perovskite Is Hampered by Intrinsic Quantum Confinement

**DOI:** 10.1021/acsenergylett.3c00656

**Published:** 2023-05-10

**Authors:** Karim A. Elmestekawy, Benjamin M. Gallant, Adam D. Wright, Philippe Holzhey, Nakita K. Noel, Michael B. Johnston, Henry J. Snaith, Laura M. Herz

**Affiliations:** † Department of Physics, University of Oxford, Clarendon Laboratory, Parks Road, Oxford OX1 3PU, United Kingdom; ‡ Institute for Advanced Study, Technical University of Munich, Lichtenbergstrasse 2a, D-85748 Garching, Germany

## Abstract

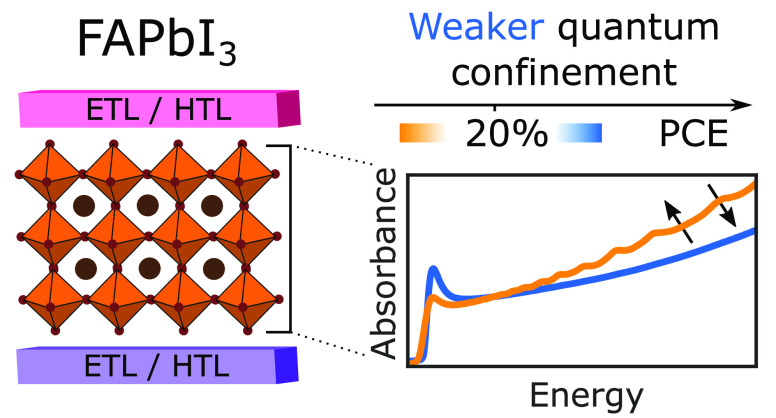

Formamidinium lead trioiodide (FAPbI_3_) is a promising perovskite for single-junction solar cells. However, FAPbI_3_ is metastable at room temperature and can cause intrinsic quantum confinement effects apparent through a series of above-bandgap absorption peaks. Here, we explore three common solution-based film-fabrication methods, neat *N*,*N*-dimethylformamide (DMF)–dimethyl sulfoxide (DMSO) solvent, DMF-DMSO with methylammonium chloride, and a sequential deposition approach. The latter two offer enhanced nucleation and crystallization control and suppress such quantum confinement effects. We show that elimination of these absorption features yields increased power conversion efficiencies (PCEs) and short-circuit currents, suggesting that quantum confinement hinders charge extraction. A meta-analysis of literature reports, covering 244 articles and 825 photovoltaic devices incorporating FAPbI_3_ films corroborates our findings, indicating that PCEs rarely exceed a 20% threshold when such absorption features are present. Accordingly, ensuring the absence of these absorption features should be the first assessment when designing fabrication approaches for high-efficiency FAPbI_3_ solar cells.

Metal halide perovskites (MHPs) have experienced tremendous progress in the past decade, reflected by a substantial improvement in the power conversion efficiency (PCE) of perovskite solar cells from a low 3.8%^1^ to values now regularly exceeding 25%^2,3^ in just over a decade. These materials owe their excellent optoelectronic properties and photovoltaic (PV) performance to benign defect chemistry,^4−10^ high charge-carrier mobilities,^11^ long diffusion lengths,^12,13^ low exciton binding energies,^14^ and broadly tunable absorption spectra.^15^ While many promising MHP compositions have been explored as active layers in photovoltaic devices, those centered around formamidinium lead triiodide (FAPbI_3_) have arguably emerged among the most promising,^16^ currently holding the single-junction MHP device efficiency record with a certified power conversion efficiency (PCE) of 25.7%^2,3,17^ and being one of the most investigated compositions according to a recent literature survey by Jacobsson et al.^18^ The success of FAPbI_3_ partly derives from its near-optimal bandgap^16^ for single-junction cells^19^ and its enhanced stability under elevated temperatures compared to other commonly explored hybrid MHPs based on methylammonium, such as MAPbI_3_.^20−22^ In addition, FAPbI_3_ importantly offers simplicity of stoichiometry compared with alloyed MHPs containing multi A-site cations or mixed halides, which enhances its resistance against compositional dealloying and the formation of compositional inhomogeneities that can adversely affect performance.^23−27^

However, one aspect of FAPbI_3_ requiring careful control is structural stability. At room temperature, the desired perovskite α-phase is only metastable and easily deteriorates into a thermodynamically favorable, yellow, nonperovskite 2H (δ)-phase that is not particularly photoactive.^28^ Early work demonstrated that partial substitution of FA with Cs with as little as 5%^28−33^ is sufficient to eliminate traces of δ-phase presence in XRD patterns,^34,35^ but such a degree of A-site alloying shifts the band gap to higher energies^32^ and has been shown to introduce undesirable compositional inhomogeneities.^36,37^ Recently, a plethora of fabrication methods and optimization approaches have therefore been developed to attain and stabilize the “unalloyed” perovskite α-phase of FAPbI_3_.^11,20,21,32,38−44^ These alterations to earlier fabrication methods^16^ vary from introducing pre- and postannealing steps and additive treatments^45,46^ to more elaborate stoichiometric engineering techniques,^40,47,48^ vapor codeposition,^43^ and more recently templating and sequential deposition strategies via solution^49^ or vapor deposition^44^ routes, yielding significant advances in stability and performance.

In addition, one peculiarity specific to FAPbI_3_ is its propensity to exhibit intrinsic quantum confinement, which has been attributed to the formation of intrinsic nanostructured domains on the length scale of ∼10 nm in subvolumes of nominally bulk films.^50,51^ Evidence for these features has been derived from the presence of peculiar above-bandgap peaks that are superimposed on bulk-like absorption spectra and that had gone mostly unnoticed and unexplained^16,32,52,53^ until recently.^50,51^ Analysis of these absorption peak features has directly pointed toward the presence of electronic quantum confinement, and while these structures have not been imaged directly to date, it has been suggested that they could derive from the presence of thin slices of α-phase FAPbI_3_ intrinsically self-assembling to form superlattices or quantum wells surrounded by regions acting as energetic barriers,^50^ potentially in an attempt to alleviate some of the experienced strain in the lattice.^54^ Such energy barriers may constitute inclusions of the δ- or alternative secondary phases forming thin layers undetectable in XRD patterns, with a relatively high Cs content of ≥40% recently having been found to eliminate any traces of such quantum confinement.^51^ It should be expected that any form of quantum confinement experienced by charge carriers even in small subvolumes of the absorber layers hinders the unobstructed passage of the photocurrent throughout a solar cell. Surprisingly though, the relationship between the presence of intrinsic quantum confinement features in the absorption spectra of FAPbI_3_ and the performance of photovoltaic devices based on these layers has not yet been examined. Such an investigation is particularly urgent in the context of the myriad of fabrication protocols developed for FAPbI_3_, which currently focus relatively narrowly on elimination of δ-phase features in XRD patterns that has, however, been shown to be an inaccurate indicator^50,51^ of whether or not intrinsic quantum confinement will be present.

In this letter, we unravel the extent to which intrinsic quantum confinement in FAPbI_3_ affects solar cell performance. We contrast evidence of quantum confinement gathered from features in thin-film optical absorption spectra with photovoltaic performance parameters extracted from devices incorporating the same absorber layers. We directly examine three different fabrication routes as exemplifiers, but to ensure that our findings are generally applicable, we also perform a comprehensive meta-analysis of literature reports across the field. While our three experimentally fabricated FAPbI_3_ films appear to be compositionally identical, the materials have experienced different crystallization dynamics and therefore are likely to possess different strain environments. Using structural and optical analysis, we show that such variations strongly affect the extent to which the FAPbI_3_ films exhibit domains experiencing intrinsic quantum confinement. We demonstrate a clear positive correlation between the photovoltaic power conversion efficiency and steady-state short-circuit current *J*_SC_ values of corresponding devices with the absence of peak features in the absorption spectrum, revealing that intrinsic quantum confinement in FAPbI_3_ hinders photovoltaic performance. In our meta-analysis of literature reports, we examine photovoltaic PCE data reported for 825 devices with three-dimensional stoichiometric FAPbI_3_ films as absorber layers across 244 literature articles and categorize these according to whether or not intrinsic quantum confinement is evident in thin-film absorption spectra reported alongside. We discover that elimination of these features is highly correlated with the recent performance gains of FAPbI_3_ solar cells. Importantly, we find that devices incorporating films that possess such features rarely exceed PCE value of 20%, suggesting that such intrinsically formed confinement domains provide inherent barriers that become clearly apparent once all other detriments have been eliminated. Accordingly, we propose that checking for the absence of these absorption features should be the first point of investigation when gauging the effectiveness of a new fabrication approach for FAPbI_3_ thin films designed to act as photoabsorber layers in high-efficiency solar cells.

We focus our experimental investigation on stoichiometric FAPbI_3_ films fabricated using three different solution-based processing routes. The first set of FAPbI_3_ films (labeled “DMF-DMSO”) were fabricated using the standard deposition method based on neat DMF-DMSO (DMF, *N*,*N*-dimethylformamide; DMSO, dimethyl sulfoxide) solvent utilizing “antisolvent quenching” without any additives aimed at stabilizing the α-phase or slowing down the crystallization process.^16,20,21,42^ The second set of FAPbI_3_ films (labeled “MACl route”) resulted from DMF-DMSO solution-casting involving the use of excess MACl (methylammonium chloride) as an additive to induce an intermediate phase which is easily transformed to the neat α-phase.^55^ MACl thus effectively directs the crystallization process by providing an alternative crystallization pathway and improving by improving the grain size and crystallographic purity of the fabricated film.^20^ Use of this additive has frequently been reported in the literature to stabilize the α-phase and consistently results in devices with enhanced PV performances, with fabrication methods now optimized to ensure the predominant exclusion of MA^+^ and Cl^–^ ions from the crystal structure in order to yield near-stoichiometric FAPbI_3_ films.^20,21,42,55−57^ The third set of FAPbI_3_ films (labeled “Sequential”) was fabricated through a sequential deposition method based on work by Noel et al.^58^ whereby first a 2D perovskite material is deposited, thermally treated, and subsequently converted to the α-FAPbI_3_ phase by means of an alcoholic solution of formamidinium iodide, analogous to archetypical two-step processing methods of halide perovskites.^49,59^ Such sequential deposition allows for careful curation of a solid-state intermediate phase and initiates the formation of high-quality FAPbI_3_ in its black perovskite α-phase, suppressing inclusions of unintended structures.^42,49,58−60^ Careful control over the emerging crystal structure can therefore be attained, which provides the additional opportunity to investigate films fabricated through a significantly more controlled route compared to those of the FAPbI_3_ films fabricated from neat DMF-DMSO solvent or even MACl-additive routes.^58^ Further details about each individual fabrication method are presented in the Supporting Information (SI).

Differences in film fabrication methods will directly influence the crystallization dynamics and structural properties of the resulting perovskite,^21,32,38−42^ which in turn will alter the optoelectronic and photovoltaic performance. We therefore begin by examining the recorded X-ray diffraction (XRD) patterns of the FAPbI_3_ films, with Figure 1a showing the relevant region of interest (full XRD patterns displayed in SI, Figure 1). The good coincidence of the main pseudocubic (100) and (200) perovskite peak angles across the three different fabrication methods and their agreement with the expected literature value for α-FAPbI_3_,^61^ highlight that they indeed yield compositionally similar stoichiometric FAPbI_3_ perovskite, without remnant inclusions of MA or Cl. However, differences between these fabrication methods can still be clearly observed in the XRD patterns. The full widths at half-maxima (fwhm) of the main (100) and (200) perovskite peaks (displayed in SI, Figure 2) reflect the longer-range crystalline order and/or lower microstrain in FAPbI_3_ films fabricated from the MACl route and sequential deposition method, compared with those for the neat DMF-DMSO route. The scattering intensity of the main α-FAPbI_3_ phase is orders of magnitude stronger for the MACl and sequential-deposition processed films, indicating increased texture. The XRD patterns further show a relatively small contribution from impurity phases such as PbI_2_ and δ-phase FAPbI_3_ (data are plotted on a logarithmic scale) that seem to show little systematic variation between processing routes, with the films resulting from sequential deposition exhibiting the highest scattering intensity for PbI_2_ and those derived from the MACl route the highest scattering intensity for the δ-phase FAPbI_3_. We note, however, that the presence of such features has been found to correlate poorly with the strength of absorption peak features, likely because the potential structural barriers causing these effects are too thin to be detectable in XRD.^50^ To further interrogate this apparent increase in the texturing of the films using the MACl route and the sequential deposition approach, we also conducted GIWAXS measurements, which we show in SI, Figure 3, along with a detailed comparative analysis of the structural properties and crystallographic quality of these differently fabricated films in SI, section 3. These data confirm an increase in crystallinity and a higher degree of orientation in films fabricated through additive-based or sequential deposition approaches.

**Figure 1:**
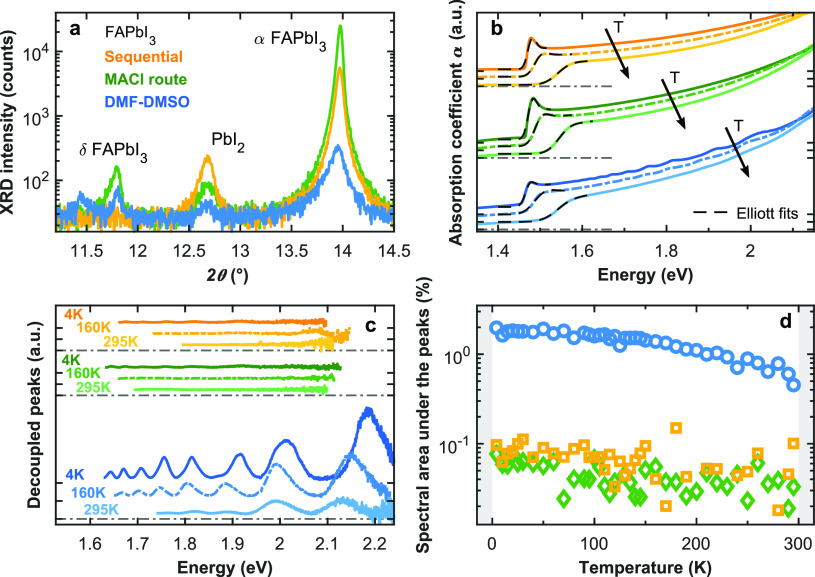
Structural and optical properties of stoichiometric FAPbI_3_ films made from different fabrication methods. (a) Excerpts from recorded X-ray diffraction (XRD) patterns focusing on the pseudocubic (100) peak of α-FAPbI_3_, the main peaks of the nonperovskite δ-FAPbI_3_ phase,^61^ and the peak of PbI_2_ precursor residue.^62^ Full XRD patterns are displayed in SI, Figure 1. (b) Absorption coefficient spectra (colored solid lines) and Elliott fits to the absorption onsets (dashed black lines) of FAPbI_3_ films fabricated via the “DMF-DMSO” (blue), “MACl route” (green), and “sequential” (yellow) solvent-based methods, recorded at 4, 160, and 295 K, respectively. The spectra recorded at different temperatures and for the different fabrication methods are vertically offset for visual clarity, with the black *y*-axis ticks marking the zero baseline for each successive curve. A direct comparison of the absorption coefficient spectra is presented in SI, Figure 7. (c) Peak features decoupled from the underlying, bulk-like absorption spectrum at 4, 160, and 295 K presented on the same scale. The spectra at different temperatures and for the different fabrication methods are vertically offset for visual clarity, with the black *y*-axis ticks marking the zero baseline for each successive curve. (d) Spectrally integrated area underneath the absorption peaks shown in (c), given as a percentage of the total area under the absorption coefficient spectrum, used to parametrize the occurrence of quantum confinement, and shown as a function of temperature for the differently fabricated FAPbI_3_ films. (The fabrication method legend in (a) also applies to (b), (c), and (d), and the temperature legend in (c) also applies to (b)).

We proceeded by probing how the three different fabrication approaches affect the occurrence of intrinsic quantum confinement in FAPbI_3_ films. For this purpose, we measured the absorption coefficient spectra at a range of different temperatures from 295 K down to 4 K (displayed in Figure 1b) in order to determine the fraction of the absorption spectrum that exhibits the peak features that have been associated with such confinement in the past.^50,51^ While cryogenic temperatures are less relevant to photovoltaic operation, it has recently been shown that they enhance the sharpness and amplitudes of the absorption features, making them easier to discern and evaluate accurately.^50,51^ To analyze the absorption coefficient spectra, we first fitted the onset with Elliott’s theory, which takes into account the excitonic contribution and Coulombic enhancement to the absorption spectrum^63,64^ (see dashed lines in Figure 1b). These fits yielded parameters such as the optical bandgap and exciton binding energy (see SI, Figure 8) that are very similar in both value and temperature trends for the films made through the three fabrication routes, further highlighting that these films are compositionally similar. Similarly, room-temperature PL peak energies are found to be identical for all three types of FAPbI_3_ films (see SI, Figure 9). As a second step, to determine the contribution to the absorption spectrum arising from quantum confinement, we decoupled the above-bandgap absorption peak features from the underlying, smooth, bulk-like spectrum using the process previously reported.^50,51^ We chose a phenomenological spline baseline connecting all the troughs of the features together and subtracted this baseline from the full absorption spectrum. The resulting decoupled peaks associated with intrinsic quantum confinement are shown in Figure 1c for different FAPbI_3_ film fabrication methods and temperatures.^50,51^ As a numerical parameter characterizing the relative prominence of quantum confinement, we evaluated the area between the experimental absorption coefficient spectrum and the spline baseline connecting the troughs (i.e., the integral over the decoupled peak spectra such as those shown in Figure 1c) as a percentage of the overall area under the absorption spectrum. This “spectral area under the peaks” is shown in Figure 1d as a function of temperature for the FAPbI_3_ films processed by the three different methods.

Intriguingly, this analysis reveals that the fabrication method has a profound influence on the prominence of the absorption peak features, despite these all being stoichiometrically similar FAPbI_3_ films. While FAPbI_3_ films fabricated from the neat DMF-DMSO method exhibit very prominent absorption peak features, those features are over an order of magnitude lower in amplitude and hardly discernible for the films fabricated through the MACl and sequential deposition routes, even at very low temperatures down to 4 K (Figure 1c,d). These differences clearly point toward the crystallization process, the experienced strain in the system, and/or small inclusions of δ-phase or one of its polymorphs playing a significant role in the self-assembly of domains causing electronic confinement in the perovskite film. Such variations make sense in the context of quantum confinement in FAPbI_3_ having been proposed to be caused by inclusions of thin layers of δ-phase that act as electronic barrier to the α-phase, possibly to achieve periodic strain relief in the system.^50^ Similarly, a range of different hexagonal polytypes have been shown to create peaked absorption features at various energies.^65^ The presence of these peaked absorption features in FAPbI_3_ therefore appear to be a direct indicator of crystalline quality. The comparatively uncontrolled crystallization process associated with the neat DMF-DMSO fabrication method is known to result in poorer crystallographic quality of FAPbI_3_ films and an instability toward reversion into the δ-phase,^11,20,21,40,42^ and these films exhibit strong absorption peak features. In contrast, the MACl additive provides intermediate-phase directed crystallization, which yields better control over growth dynamics, while the sequential deposition method nucleates α-phase growth, both of which lead to superior crystalline quality, and, as we show here, a significantly lower propensity toward formation of inferred quantum-confined domains.^55,66^ We therefore demonstrate that thin-film nucleation and crystallization control can regulate and eliminate the quantum confinement experienced in FAPbI_3_.

The appearance of domains exhibiting electronic confinement has the potential to hinder charge-carrier extraction in solar cells. We thus proceeded by assessing the extent to which the presence of such absorption features affects the performance of photovoltaic devices incorporating FAPbI_3_ absorber layers fabricated using the neat DMF-DMSO, MACl, and sequential deposition routes. Devices were fabricated using a typical n-i-p configuration (FTO/SnO_2_/FAPbI_3_/PEAI/spiro-OMeTAD/Au). Here, the main difference in PV performance parameters should therefore be associated with the properties of each FAPbI_3_ photoabsorber layer. Accordingly, the steady-state short-circuit current density (*J*_SC_) of optimized devices can be treated as a parameter to gauge how efficient and unobstructed the charge-carrier motion is throughout the MHP layer. Full details of device fabrication and more detailed comparisons of the performance parameters for these devices are presented in section 7 in the SI. In addition, for each FAPbI_3_ film fabrication type, absorber layer thicknesses were optimized for best performance, yielding 340, 440, and 770 nm for the neat DMF-DMSO, MACl, and sequential deposition routes, respectively (see SEM images in SI, section 4, Figures 4–6).^58^ While we acknowledge that generally, increasing the thickness of the perovskite layer will result in increased light absorption and hence, current generation, we note that the devices made with FAPbI_3_ films of similar thicknesses, processed with and without the MACl additive, still possessed a substantial difference in their steady-state *J*_SC_ values (see SI, section 7). As such, we conclude that the thickness variations in the optimized devices do not affect our general conclusions.

A direct comparison of photovoltaic performance parameters for devices incorporating stoichiometric FAPbI_3_ films made with the three different fabrication methods is displayed in Figure 2 and SI, Figures 10–13. The PCEs and steady-state *J*_SC_ values exhibit a notable improvement for FAPbI_3_ films fabricated through the MACl route and sequential deposition method compared with the neat DMF-DMSO method. We note that the MACl route and sequential deposition procedure have been shown to result in improved interfacial alignment with the employed transport layers and trap passivation^67−69^ that are likely to be responsible for the slight increase in steady-state open-circuit voltage (*V*_OC_) of the corresponding devices we observe here (Figure 2b). However, such improvements would not be expected to also cause a significant enhancement to the charge-carrier extraction capabilities, and yet, devices incorporating films fabricated through the MACl and sequential deposition routes possess a substantially higher steady-state *J*_SC_ than those made from the neat DMF-DMSO protocol. Instead, these improvements in steady-state *J*_SC_ with the MACl route and sequential deposition approach appear to be directly correlated with the removal of the absorption peak features for these types of absorber layers we reported above. Such quantum confinement domains thus appear to induce significantly lower stabilsied *J*_SC_ as a clear indication of poor charge-carrier extraction, consistent with the emergence of high-energy potential barriers obstructing transport across the film.

**Figure 2:**
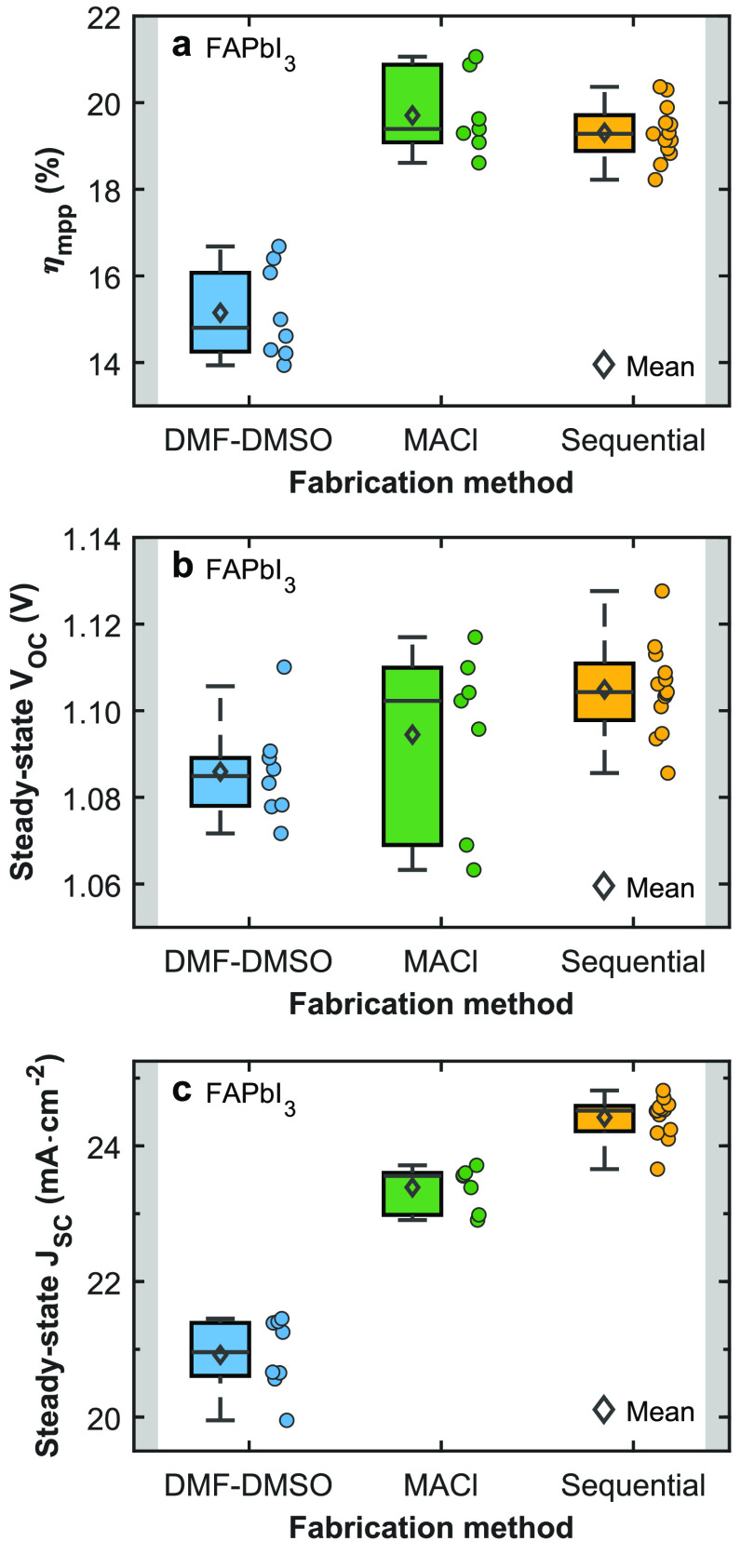
Performance parameters for photovoltaic devices incorporating FAPbI_3_ films made from different fabrication methods. Box and scatter plots showing the steady-state photovoltaic parameters for devices based on FAPbI_3_ absorber layers fabricated through the “DMF-DMSO” (blue), “MACl route” (green), and “sequential” (yellow) solvent-based methods. (a) Maximum power point tracked power conversion efficiency (η_mpp_). (b) Steady-state open-circuit voltage (*V*_OC_). (c) Steady-state short-circuit current density *J*_SC_. The scatter points represent the different device pixels investigated. A comparison of the performance parameters for photovoltaic devices incorporating FAPbI_3_ films of different thicknesses fabricated via the “DMF-DMSO” route is displayed in SI, Figure 14.

Our analysis suggests that the quantum confinement present in FAPbI_3_-rich films heavily depends on the fabrication method and is detrimental to the performance of the associated photovoltaic device. To examine whether these findings hold more generally beyond the investigated fabrication methods, we conducted a meta-analysis across a wide range of literature reports. The meta-study was carried out by correlating reported PCE values of FAPbI_3_ solar cells with the presence or absence of quantum confinement, as determined from the analysis described and used above of the presence of peak features in the absorption spectra of corresponding films. Selection was effectively limited to devices whose photoactive layer was reported to be FAPbI_3_ three-dimensional perovskite thin films, i.e., we excluded any reports where additives or treatments were proven to be fully incorporated into the crystal structure such that they changed the stoichiometry. As a result, 244 unique publications were included in the meta-study, and a total of 825 device performance reports, providing statistically significant correlation data between PV performance and the presence of quantum confinement (QC). The PV performance data were either the batch-averages across many reported devices, or data corresponding to the champion cell if an average was not reported. Overall, the very large number of devices and published reports on the FAPbI_3_ composition (see also Figure 3j) is directly reflective of the historical and continued interest in this thermally stable composition. To assess the correlation between photovoltaic performance and the presence of quantum confinement, we divided the parameters associated with different devices into three unique categories. First, solar cells whose corresponding photoabsorber layer either possessed easily discernible above-bandgap absorption features or features above a certain minimum threshold were labeled “QC present” and amounted to 100 devices. Second, solar cells with no apparent visible features or features below the threshold were labeled “QC absent” and amounted to 302 devices. Finally, when absorption spectra were absent in the publication reporting the PV performance, or were too noisy to allow conclusion of presence or absence of features, this was labeled “QC data unavailable” and amounted to 423 devices. We note that only about half (48.7%) of the reported device performance parameters come with sufficient absorption data. While this still provides us with ample available data (402 device reports) to retrieve statistically significant correlations, it highlights that insufficient attention has been devoted to these absorption features and their relevance to photovoltaic device performance to date. Full details on the nuances of our meta-study can be found in SI, section 8.

**Figure 3:**
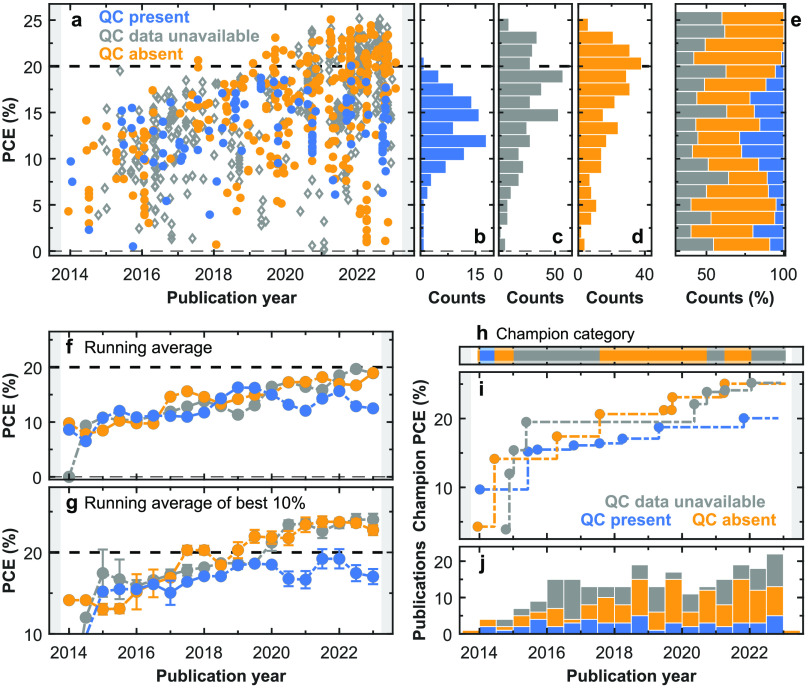
Meta-analysis of photovoltaic device performance and presence of absorbance features for FAPbI_3_ thin films extracted from literature. (a) Reported PCE values of single-junction solar cells whose photoabsorber layers compromise stoichiometric bulk FAPbI_3_ thin films, plotted as a function of publication year, visualized for three categories of FAPbI_3_ films; films with above-bandgap “quantum confinement (QC)” absorption features present (labeled as “QC present”, blue), films for which absorption spectra were unavailable or of insufficient quality or spectral range (labeled as “QC data unavailable”, gray), and films for which absorption data showed no discernible peak features, or features below a certain low threshold (labeled as “QC absent”, orange); see SI, section 8, for full details. (b–d) The total time-integrated device count for each PCE range across the “QC present”, “QC data unavailable”, and “QC absent” categories, respectively. (e) The relative contribution of each category to the available device data for every PCE range, integrated over the years of publication. (f,g) Running average of PCE values recorded for all devices reported in literature, and for the best 10% of devices, respectively, for each category with an averaging window of one year. (h) The time evolution of the absorber film category associated with the highest PCE FAPbI_3_ devices. (i) The time evolution of the highest PCE device for each absorber film category. (j) Distribution of the number of unique publications with data on solar cell performance for FAPbI_3_ films as a function of publication year divided into the three absorber film categories. The color legend in (a) also applies to b–j).

The results from our meta-analysis are visualized in a range of different scatter plots in Figure 3 and SI, Figures 15–24. At first sight, it is apparent that PCE values of FAPbI_3_ solar cells follow a clear upward trajectory of continuous enhancement of PV properties over time, culminating in the highest certified PCE for a MHP single junction of 25.7%^3,17^ for this composition (Figure 3a). Examining device performance according to the three categories of quantum confinement (present, absent, data unavailable), it also becomes clear that such QC effects have been present or absent since the first realization of working FAPbI_3_ PV devices, and that the simple running average of the PCE follows a similar upward trajectory (see Figure 3f). This unrelenting enhancement demonstrates the intense interest invested by the field in this composition and highlights the success of current research in tackling some major hurdles such as reducing defect concentration, improving crystallinity, interface quality, and charge extraction layers. However, more recently, noticeable differences have started to emerge as devices are breaking through the 20% PCE ceiling, reflective of a region where quantum confinement appears to become the decisive factor.

Such evidence for QC features in absorption restricting performance near the Shockley–Queisser limit can be seen in the meta-data in a number of forms. First, Figure 3a shows that since 2019, any meaningful progress in PCE values of devices in the “QC present” category (blue dots) has stalled, with a maximum of only 20.05% attained, compared with 25.06% for the “QC absent” and 25.18% for the “QC data unavailable” categories, suggesting that an intrinsic barrier exists for efficient charge-carrier extraction and PV performance for devices employing films possessing these peculiar QC features. The time-integrated PCE spread in Figure 3b,c,d clearly highlights an abundance of devices in the “QC absent” and “QC data unavailable” categories, with PCE values regularly exceeding the 20% mark, while devices in the “QC present” category very rarely approach that threshold. This is further illustrated in Figures 3h,i, which show that both the “QC absent” and “QC data unavailable” categories also dominate the champion performance since 2014. Finally, Figure 3g provides a temporal evolution of the running average of the best 10% of all devices within each of the three categories showing that even for such averages across the top end of the performance spectrum, the “QC absent” and “QC data unavailable” categories clearly outrun the performance of devices in the “QC present” category. Overall, these meta-data clearly reflect the stagnation in the PV enhancement of the devices in the “QC present” category and the futility of attempts to improve the PCE of a device beyond 20% without attempting to suppress these absorption peak features. Based on this established correlation, a necessary but insufficient condition to obtain devices with efficiencies exceeding ∼20% is to ensure the absence of any above-bandgap QC features in the absorption spectrum. We note that, in fact, most of the recent literature-reported attempts to alter the fabrication method of this promising composition with the aim of optimizing its photovoltaic performance have inadvertently been suppressing these peculiar absorption features.^38,47,57,70−79^ However, the lack of comment on such features in literature reports, and an absence of absorption spectra in over half of the reports we examined, clearly show that little attention has to date been paid to this simple design and validation criterion. Accordingly, we propose that a first point for investigating a new fabrication protocol for FAPbI_3_ films to be incorporated into solar cells should be to discern the presence or absence of these peak features in the thin-film absorption spectrum. Such simple assessment can ascertain whether there is a tendency for the fabricated FAPbI_3_ films to include electronic barriers that are too thin to be detectable in XRD patterns, but that may still inhibit charge-carrier extraction and whose elimination will thus pave the way for high-performance solar cells.

In conclusion, our work has shown that to realize the highest performing solar cells based on the attractive FAPbI_3_ perovskite, quantum confinement needs to be fully eliminated from within the thin-film layer, most likely because it impedes charge-carrier transport and hence restrains the maximum attainable steady-state *J*_SC_ values. We have conclusively demonstrated this finding through two approaches. First, we have examined a set of stoichiometric FAPbI_3_ films with varying degrees of quantum confinement induced by means of three different solution-based film fabrication processes. Those methods capable of reducing the strain, directing and controlling crystallization, and thus increasing perovskite crystalline quality strongly suppressed the quantum confinement features and clearly enhanced the solar cell performance parameters, in particular, obtainable steady-state *J*_SC_ values. Second, we have performed a meta-analysis across 244 publications, analyzing 825 occurrences of PCE data from FAPbI_3_ solar cells in order to search for correlations with the presence or absence of the quantum confinement features evident from peaks in the matching absorption spectra. Our meta-study reveals that for FAPbI_3_ solar cells to break through the 20% PCE ceiling, quantum confinement effects should be absent from the film.

These conclusions clearly highlight simple checks for peaked absorption features as an easy step in the development of new thin-film fabrication procedures aimed at delivering efficient photovoltaic cells based on FAPbI_3_. We note that the presence or absence of phase impurities, such as the δ-phase, in XRD patterns is unlikely to be as conclusive because even FAPbI_3_ films with strong absorption peak features have at times been found to display no discernible δ-phase peak in XRD.^50^ These electronic barriers may thus offer too few lattice planes to allow sufficiently sharp deflection of X-rays, leading to insufficiently sharp XRD peaks that cannot easily be detected. The alternative criterion we propose here, i.e., an examination of the absorption spectrum, is a simple and experimentally straightforward approach already accessible in the vast majority of research laboratories. This approach should therefore be an easy and powerful means to gauge the effectiveness of any newly developed treatment method for FAPbI_3_ thin-film absorber layers. Overall, our findings will accelerate power conversion efficiencies of the most commercially viable MHP on the last stretch toward the maximum theoretical attainable efficiencies for single-junction photovoltaic devices.

## Supporting Information Available

Fabrication method, experimental details, XRD patterns and analysis, cross-sectional scanning electron microscopy images, additional absorption coefficient spectra and details on their fits, additional photovoltaic device performance measurements, and details on the meta-study and subsequent analysis (PDF)

## References

[ref1] Kojima A.; Teshima K.; Shirai Y.; Miyasaka T. Organometal halide perovskites as visible-light sensitizers for photovoltaic cells. J. Am. Chem. Soc. 2009, 131, 6050–6051. 10.1021/ja809598r. 19366264

[ref2] Green M. A.; Dunlop E. D.; Hohl-Ebinger J.; Yoshita M.; Kopidakis N.; Bothe K.; Hinken D.; Rauer M.; Hao X. Solar cell efficiency tables (Version 60). Progress in Photovoltaics: Research and Applications 2022, 30, 687–701. 10.1002/pip.3595.

[ref3] Park J.; Kim J.; Yun H.-S.; Paik M. J.; Noh E.; Mun H. J.; Kim M. G.; Shin T. J.; Seok S. I. Controlled growth of perovskite layers with volatile alkylammonium chlorides. Nature 2023, 616, 724–730. 10.1038/s41586-023-05825-y. 36796426

[ref4] Herz L. M. Charge-carrier mobilities in metal halide perovskites: fundamental mechanisms and limits. ACS Energy Letters 2017, 2, 1539–1548. 10.1021/acsenergylett.7b00276.

[ref5] Yin W.-J.; Shi T.; Yan Y. Unusual defect physics in CH_3_NH_3_PbI_3_ perovskite solar cell absorber. Appl. Phys. Lett. 2014, 104, 063903 10.1063/1.4864778.

[ref6] Kim J.; Lee S.-H.; Lee J. H.; Hong K.-H. The role of intrinsic defects in methylammonium lead iodide perovskite. J. Phys. Chem. Lett. 2014, 5, 1312–1317. 10.1021/jz500370k. 26269973

[ref7] Azpiroz J. M.; Mosconi E.; Bisquert J.; De Angelis F. Defect migration in methylammonium lead iodide and its role in perovskite solar cell operation. Energy Environ. Sci. 2015, 8, 2118–2127. 10.1039/C5EE01265A.

[ref8] Yin W.-J.; Shi T.; Yan Y. Unique properties of halide perovskites as possible origins of the superior solar cell performance. Adv. Mater. 2014, 26, 4653–4658. 10.1002/adma.201306281. 24827122

[ref9] Stoumpos C. C.; Kanatzidis M. G. The renaissance of halide perovskites and their evolution as emerging semiconductors. Acc. Chem. Res. 2015, 48, 2791–2802. 10.1021/acs.accounts.5b00229. 26350149

[ref10] Stoumpos C. C.; Kanatzidis M. G. Halide Perovskites: poor Man’s high-performance semiconductors. Adv. Mater. 2016, 28, 5778–5793. 10.1002/adma.201600265. 27174223

[ref11] Rehman W.; McMeekin D. P.; Patel J. B.; Milot R. L.; Johnston M. B.; Snaith H. J.; Herz L. M. Photovoltaic mixed-cation lead mixed-halide perovskites: links between crystallinity, photo-stability and electronic properties. Energy Environ. Sci. 2017, 10, 361–369. 10.1039/C6EE03014A.

[ref12] Wehrenfennig C.; Eperon G. E.; Johnston M. B.; Snaith H. J.; Herz L. M. High charge carrier mobilities and lifetimes in organolead trihalide perovskites. Adv. Mater. 2014, 26, 1584–1589. 10.1002/adma.201305172. 24757716 PMC4722848

[ref13] Stranks S. D.; Eperon G. E.; Grancini G.; Menelaou C.; Alcocer M. J.; Leijtens T.; Herz L. M.; Petrozza A.; Snaith H. J. Electron-hole diffusion lengths exceeding 1 micrometer in an organometal trihalide perovskite absorber. Science 2013, 342, 341–344. 10.1126/science.1243982. 24136964

[ref14] Herz L. M. Charge-carrier dynamics in organic-inorganic metal halide perovskites. Annu. Rev. Phys. Chem. 2016, 67, 65–89. 10.1146/annurev-physchem-040215-112222. 26980309

[ref15] Green M. A.; Ho-Baillie A.; Snaith H. J. The emergence of perovskite solar cells. Nat. Photonics 2014, 8, 506–514. 10.1038/nphoton.2014.134.

[ref16] Eperon G. E.; Stranks S. D.; Menelaou C.; Johnston M. B.; Herz L. M.; Snaith H. J. Formamidinium lead trihalide: a broadly tunable perovskite for efficient planar heterojunction solar cells. Energy Environ. Sci. 2014, 7, 982–988. 10.1039/c3ee43822h.

[ref17] Best Research-Cell Efficiency Chart; NREL, 2022; https://www.nrel.gov/pv/cell-efficiency.html (accessed 2022-08-23).

[ref18] Jacobsson T. J.; Hultqvist A.; García-Fernández A.; Anand A.; Al-Ashouri A.; Hagfeldt A.; Crovetto A.; Abate A.; Ricciardulli A. G.; Vijayan A.; et al. An open-access database and analysis tool for perovskite solar cells based on the FAIR data principles. Nature Energy 2022, 7, 107–115. 10.1038/s41560-021-00941-3.

[ref19] Shockley W.; Queisser H. J. Detailed balance limit of efficiency of p–n junction solar cells. J. Appl. Phys. 1961, 32, 510–519. 10.1063/1.1736034.

[ref20] An Y.; Hidalgo J.; Perini C. A. R.; Castro-Mendez A.-F.; Vagott J. N.; Bairley K.; Wang S.; Li X.; Correa-Baena J.-P. Structural Stability of Formamidinium-and Cesium-Based Halide Perovskites. ACS Energy Letters 2021, 6, 1942–1969. 10.1021/acsenergylett.1c00354.

[ref21] Lee J.-W.; Tan S.; Seok S. I.; Yang Y.; Park N.-G. Rethinking the A cation in halide perovskites. Science 2022, 375, eabj1186 10.1126/science.abj1186. 35201885

[ref22] Conings B.; Drijkoningen J.; Gauquelin N.; Babayigit A.; D’Haen J.; D’Olieslaeger L.; Ethirajan A.; Verbeeck J.; Manca J.; Mosconi E.; et al. Intrinsic thermal instability of methylammonium lead trihalide perovskite. Adv. Energy Mater. 2015, 5, 1500477 10.1002/aenm.201500477.

[ref23] Knight A. J.; Herz L. M. Preventing phase segregation in mixed-halide perovskites: a perspective. Energy Environ. Sci. 2020, 13, 2024–2046. 10.1039/D0EE00788A.

[ref24] Bercegol A.; Ramos F. J.; Rebai A.; Guillemot T.; Puel J.-B.; Guillemoles J.-F.; Ory D.; Rousset J.; Lombez L. Spatial inhomogeneity analysis of cesium-rich wrinkles in triple-cation perovskite. J. Phys. Chem. C 2018, 122, 23345–23351. 10.1021/acs.jpcc.8b07436.

[ref25] Chatterjee R.; Pavlovetc I. M.; Aleshire K.; Hartland G. V.; Kuno M. Subdiffraction infrared imaging of mixed cation perovskites: Probing local cation heterogeneities. ACS Energy Letters 2018, 3, 469–475. 10.1021/acsenergylett.7b01306.

[ref26] Barrier J.; Beal R. E.; Gold-Parker A.; Vigil J. A.; Wolf E.; Waquier L.; Weadock N. J.; Zhang Z.; Schelhas L. T.; Nogueira A. F.; et al. Compositional heterogeneity in Cs_*y*_FA_1–*y*_Pb(Br_*x*_I_1–*x*_)_3_ perovskite films and its impact on phase behavior. Energy Environ. Sci. 2021, 14, 6394–6405. 10.1039/D1EE01184G.

[ref27] Knight A. J.; Borchert J.; Oliver R. D.; Patel J. B.; Radaelli P. G.; Snaith H. J.; Johnston M. B.; Herz L. M. Halide segregation in mixed-halide perovskites: influence of A-site cations. ACS Energy Letters 2021, 6, 799–808. 10.1021/acsenergylett.0c02475. 33614967 PMC7888268

[ref28] Chen T.; Foley B. J.; Park C.; Brown C. M.; Harriger L. W.; Lee J.; Ruff J.; Yoon M.; Choi J. J.; Lee S.-H. Entropy-driven structural transition and kinetic trapping in formamidinium lead iodide perovskite. Science Advances 2016, 2, e1601650 10.1126/sciadv.1601650. 27819055 PMC5088641

[ref29] Eperon G. E.; Beck C. E.; Snaith H. J. Cation exchange for thin film lead iodide perovskite interconversion. Materials Horizons 2016, 3, 63–71. 10.1039/C5MH00170F.

[ref30] Saliba M.; Matsui T.; Seo J.-Y.; Domanski K.; Correa-Baena J.-P.; Nazeeruddin M. K.; Zakeeruddin S. M.; Tress W.; Abate A.; Hagfeldt A.; et al. Cesium-containing triple cation perovskite solar cells: improved stability, reproducibility and high efficiency. Energy Environ. Sci. 2016, 9, 1989–1997. 10.1039/C5EE03874J. 27478500 PMC4936376

[ref31] Yi C.; Luo J.; Meloni S.; Boziki A.; Ashari-Astani N.; Grätzel C.; Zakeeruddin S. M.; Röthlisberger U.; Grätzel M. Entropic stabilization of mixed A-cation ABX_3_ metal halide perovskites for high performance perovskite solar cells. Energy Environ. Sci. 2016, 9, 656–662. 10.1039/C5EE03255E.

[ref32] Lee J.-W.; Kim D.-H.; Kim H.-S.; Seo S.-W.; Cho S. M.; Park N.-G. Formamidinium and cesium hybridization for photo-and moisture-stable perovskite solar cell. Adv. Energy Mater. 2015, 5, 1501310 10.1002/aenm.201501310.

[ref33] Li Z.; Yang M.; Park J.-S.; Wei S.-H.; Berry J. J.; Zhu K. Stabilizing perovskite structures by tuning tolerance factor: formation of formamidinium and cesium lead iodide solid-state alloys. Chem. Mater. 2016, 28, 284–292. 10.1021/acs.chemmater.5b04107.

[ref34] Masi S.; Gualdrón-Reyes A. F.; Mora-Sero I. Stabilization of Black Perovskite Phase in FAPbI_3_ and CsPbI_3_. ACS Energy Letters 2020, 5, 1974–1985. 10.1021/acsenergylett.0c00801.

[ref35] Ghosh D.; Smith A. R.; Walker A. B.; Islam M. S. Mixed A-cation perovskites for solar cells: atomic-scale insights into structural distortion, hydrogen bonding, and electronic properties. Chem. Mater. 2018, 30, 5194–5204. 10.1021/acs.chemmater.8b01851.

[ref36] Mundt L. E.; Zhang F.; Palmstrom A. F.; Xu J.; Tirawat R.; Kelly L. L.; Stone K. H.; Zhu K.; Berry J. J.; Toney M. F.; et al. Mixing matters: nanoscale heterogeneity and stability in metal halide perovskite solar cells. ACS Energy Letters 2022, 7, 471–480. 10.1021/acsenergylett.1c02338.

[ref37] Schelhas L. T.; Li Z.; Christians J. A.; Goyal A.; Kairys P.; Harvey S. P.; Kim D. H.; Stone K. H.; Luther J. M.; Zhu K.; et al. Insights into operational stability and processing of halide perovskite active layers. Energy Environ. Sci. 2019, 12, 1341–1348. 10.1039/C8EE03051K.

[ref38] Kim G.; Min H.; Lee K. S.; Yoon S. M.; Seok S. I.; et al. Impact of strain relaxation on performance of α-formamidinium lead iodide perovskite solar cells. Science 2020, 370, 108–112. 10.1126/science.abc4417. 33004518

[ref39] Syzgantseva O. A.; Saliba M.; Grätzel M.; Rothlisberger U. Stabilization of the perovskite phase of formamidinium lead triiodide by methylammonium, Cs, and/or Rb doping. J. Phys. Chem. Lett. 2017, 8, 1191–1196. 10.1021/acs.jpclett.6b03014. 28229595

[ref40] McMeekin D. P.; Sadoughi G.; Rehman W.; Eperon G. E.; Saliba M.; Hörantner M. T.; Haghighirad A.; Sakai N.; Korte L.; Rech B.; et al. A mixed-cation lead mixed-halide perovskite absorber for tandem solar cells. Science 2016, 351, 151–155. 10.1126/science.aad5845. 26744401

[ref41] Andaji-Garmaroudi Z.; Abdi-Jalebi M.; Guo D.; Macpherson S.; Sadhanala A.; Tennyson E. M.; Ruggeri E.; Anaya M.; Galkowski K.; Shivanna R.; et al. A Highly Emissive Surface Layer in Mixed-Halide Multication Perovskites. Adv. Mater. 2019, 31, 1902374 10.1002/adma.201902374. 31489713

[ref42] Liu Z.; Liu P.; Li M.; He T.; Liu T.; Yu L.; Yuan M. Efficient and Stable FA-Rich Perovskite Photovoltaics: From Material Properties to Device Optimization. Adv. Energy Mater. 2022, 12, 2200111 10.1002/aenm.202200111.

[ref43] Borchert J.; Milot R. L.; Patel J. B.; Davies C. L.; Wright A. D.; Martinez Maestro L.; Snaith H. J.; Herz L. M.; Johnston M. B. Large-area, highly uniform evaporated formamidinium lead triiodide thin films for solar cells. ACS Energy Letters 2017, 2, 2799–2804. 10.1021/acsenergylett.7b00967.

[ref44] Lin D.; Gao Y.; Zhang T.; Zhan Z.; Pang N.; Wu Z.; Chen K.; Shi T.; Pan Z.; Liu P.; et al. Vapor Deposited Pure α-FAPbI_3_ Perovskite Solar Cell via Moisture-Induced Phase Transition Strategy. Adv. Funct. Mater. 2022, 32, 2208392 10.1002/adfm.202208392.

[ref45] McMeekin D. P.; Wang Z.; Rehman W.; Pulvirenti F.; Patel J. B.; Noel N. K.; Johnston M. B.; Marder S. R.; Herz L. M.; Snaith H. J. Crystallization Kinetics and Morphology Control of Formamidinium–Cesium Mixed-Cation Lead Mixed-Halide Perovskite via Tunability of the Colloidal Precursor Solution. Adv. Mater. 2017, 29, 1607039 10.1002/adma.201607039. 28561912

[ref46] Chen H.; Chen Y.; Zhang T.; Liu X.; Wang X.; Zhao Y. Advances to high-performance black-phase FAPbI_3_ perovskite for efficient and stable photovoltaics. Small Structures 2021, 2, 2000130 10.1002/sstr.202000130.

[ref47] Jeong J.; Kim M.; Seo J.; Lu H.; Ahlawat P.; Mishra A.; Yang Y.; Hope M. A.; Eickemeyer F. T.; Kim M.; et al. Pseudo-halide anion engineering for α-FAPbI_3_ perovskite solar cells. Nature 2021, 592, 381–385. 10.1038/s41586-021-03406-5. 33820983

[ref48] Kim M.; Jeong J.; Lu H.; Lee T. K.; Eickemeyer F. T.; Liu Y.; Choi I. W.; Choi S. J.; Jo Y.; Kim H.-B.; et al. Conformal quantum dot-SnO_2_ layers as electron transporters for efficient perovskite solar cells. Science 2022, 375, 302–306. 10.1126/science.abh1885. 35050659

[ref49] Burschka J.; Pellet N.; Moon S.-J.; Humphry-Baker R.; Gao P.; Nazeeruddin M. K.; Grätzel M. Sequential deposition as a route to high-performance perovskite-sensitized solar cells. Nature 2013, 499, 316–319. 10.1038/nature12340. 23842493

[ref50] Wright A. D.; Volonakis G.; Borchert J.; Davies C. L.; Giustino F.; Johnston M. B.; Herz L. M. Intrinsic quantum confinement in formamidinium lead triiodide perovskite. Nat. Mater. 2020, 19, 1201–1206. 10.1038/s41563-020-0774-9. 32839586

[ref51] Elmestekawy K. A.; Wright A. D.; Lohmann K. B.; Borchert J.; Johnston M. B.; Herz L. M. Controlling Intrinsic Quantum Confinement in Formamidinium Lead Triiodide Perovskite through Cs Substitution. ACS Nano 2022, 16, 9640–9650. 10.1021/acsnano.2c02970. 35609245 PMC9245356

[ref52] Lv S.; Pang S.; Zhou Y.; Padture N. P.; Hu H.; Wang L.; Zhou X.; Zhu H.; Zhang L.; Huang C.; et al. One-step, solution-processed formamidinium lead trihalide (FAPbI_(3–*x*)_Cl_*x*_) for mesoscopic perovskite-polymer solar cells. Phys. Chem. Chem. Phys. 2014, 16, 19206–19211. 10.1039/C4CP02113D. 25096582

[ref53] Pang S.; Hu H.; Zhang J.; Lv S.; Yu Y.; Wei F.; Qin T.; Xu H.; Liu Z.; Cui G. NH_2_CH = NH_2_PbI_3_: an alternative organolead iodide perovskite sensitizer for mesoscopic solar cells. Chem. Mater. 2014, 26, 1485–1491. 10.1021/cm404006p.

[ref54] McKenna K. P. Electronic properties of {111} twin boundaries in a mixed-ion lead halide perovskite solar absorber. ACS Energy Letters 2018, 3, 2663–2668. 10.1021/acsenergylett.8b01700.

[ref55] Kim M.; Kim G.-H.; Lee T. K.; Choi I. W.; Choi H. W.; Jo Y.; Yoon Y. J.; Kim J. W.; Lee J.; Huh D.; et al. Methylammonium chloride induces intermediate phase stabilization for efficient perovskite solar cells. Joule 2019, 3, 2179–2192. 10.1016/j.joule.2019.06.014.

[ref56] Wang Z.; Zhou Y.; Pang S.; Xiao Z.; Zhang J.; Chai W.; Xu H.; Liu Z.; Padture N. P.; Cui G. Additive-modulated evolution of HC(NH_2_)_2_PbI_3_ black polymorph for mesoscopic perovskite solar cells. Chem. Mater. 2015, 27, 7149–7155. 10.1021/acs.chemmater.5b03169.

[ref57] Ye F.; Ma J.; Chen C.; Wang H.; Xu Y.; Zhang S.; Wang T.; Tao C.; Fang G. Roles of MACl in Sequentially Deposited Bromine-Free Perovskite Absorbers for Efficient Solar Cells. Adv. Mater. 2021, 33, 2007126 10.1002/adma.202007126. 33296122

[ref58] Noel N. K.; Wenger B.; Habisreutinger S. N.; Snaith H. J. Utilizing nonpolar organic solvents for the deposition of metal-halide perovskite films and the realization of organic semiconductor/perovskite composite photovoltaics. ACS Energy Letters 2022, 7, 1246–1254. 10.1021/acsenergylett.2c00120. 35558900 PMC9084604

[ref59] Yang W. S.; Noh J. H.; Jeon N. J.; Kim Y. C.; Ryu S.; Seo J.; Seok S. I. High-performance photovoltaic perovskite layers fabricated through intramolecular exchange. Science 2015, 348, 1234–1237. 10.1126/science.aaa9272. 25999372

[ref60] Bu T.; Li J.; Li H.; Tian C.; Su J.; Tong G.; Ono L. K.; Wang C.; Lin Z.; Chai N.; et al. Lead halide-templated crystallization of methylamine-free perovskite for efficient photovoltaic modules. Science 2021, 372, 1327–1332. 10.1126/science.abh1035. 34140385

[ref61] Stoumpos C. C.; Malliakas C. D.; Kanatzidis M. G. Semiconducting tin and lead iodide perovskites with organic cations: phase transitions, high mobilities, and near-infrared photoluminescent properties. Inorg. Chem. 2013, 52, 9019–9038. 10.1021/ic401215x. 23834108

[ref62] Trots D.; Myagkota S. High-temperature structural evolution of caesium and rubidium triiodoplumbates. J. Phys. Chem. Solids 2008, 69, 2520–2526. 10.1016/j.jpcs.2008.05.007.

[ref63] Elliott R. Intensity of optical absorption by excitons. Phys. Rev. 1957, 108, 1384 10.1103/PhysRev.108.1384.

[ref64] Davies C. L.; Filip M. R.; Patel J. B.; Crothers T. W.; Verdi C.; Wright A. D.; Milot R. L.; Giustino F.; Johnston M. B.; Herz L. M. Bimolecular recombination in methylammonium lead triiodide perovskite is an inverse absorption process. Nat. Commun. 2018, 9, 293 10.1038/s41467-017-02670-2. 29348550 PMC5773627

[ref65] Gratia P.; Zimmermann I.; Schouwink P.; Yum J.-H.; Audinot J.-N.; Sivula K.; Wirtz T.; Nazeeruddin M. K. The many faces of mixed ion perovskites: unraveling and understanding the crystallization process. ACS Energy Letters 2017, 2, 2686–2693. 10.1021/acsenergylett.7b00981.

[ref66] Dutta N. S.; Noel N. K.; Arnold C. B. Crystalline nature of colloids in methylammonium lead halide perovskite precursor inks revealed by cryo-electron microscopy. J. Phys. Chem. Lett. 2020, 11, 5980–5986. 10.1021/acs.jpclett.0c01975. 32633521

[ref67] Byranvand M. M.; Saliba M. Defect Passivation of Perovskite Films for Highly Efficient and Stable Solar Cells. Solar RRL 2021, 5, 2100295 10.1002/solr.202100295.

[ref68] Jiang Q.; Zhao Y.; Zhang X.; Yang X.; Chen Y.; Chu Z.; Ye Q.; Li X.; Yin Z.; You J. Surface passivation of perovskite film for efficient solar cells. Nat. Photonics 2019, 13, 460–466. 10.1038/s41566-019-0398-2.

[ref69] Wolff C. M.; Caprioglio P.; Stolterfoht M.; Neher D. Nonradiative recombination in perovskite solar cells: the role of interfaces. Adv. Mater. 2019, 31, 1902762 10.1002/adma.201902762. 31631441

[ref70] Lu H.; Liu Y.; Ahlawat P.; Mishra A.; Tress W. R.; Eickemeyer F. T.; Yang Y.; Fu F.; Wang Z.; Avalos C. E.; et al. Vapor-assisted deposition of highly efficient, stable black-phase FAPbI_3_ perovskite solar cells. Science 2020, 370, eabb8985 10.1126/science.abb8985. 33004488

[ref71] Hui W.; Chao L.; Lu H.; Xia F.; Wei Q.; Su Z.; Niu T.; Tao L.; Du B.; Li D.; et al. Stabilizing black-phase formamidinium perovskite formation at room temperature and high humidity. Science 2021, 371, 1359–1364. 10.1126/science.abf7652. 33766883

[ref72] Park B.-w.; Kwon H. W.; Lee Y.; Lee D. Y.; Kim M. G.; Kim G.; Kim K.-j.; Kim Y. K.; Im J.; Shin T. J.; et al. Stabilization of formamidinium lead triiodide α-phase with isopropylammonium chloride for perovskite solar cells. Nature Energy 2021, 6, 419–428. 10.1038/s41560-021-00802-z.

[ref73] Zhu J.; Park S.; Gong O. Y.; Sohn C.; Li Z.; Zhang Z.; Jo B.; Kim W.; Han G. S.; Kim D. H.; et al. Formamidine disulfide oxidant as a localised electron scavenger for &gt;20% perovskite solar cell modules. Energy Environ. Sci. 2021, 14, 4903–4914. 10.1039/D1EE01440D.

[ref74] Jeong S.; Seo S.; Yang H.; Park H.; Shin S.; Ahn H.; Lee D.; Park J. H.; Park N.-G.; Shin H. Cyclohexylammonium-Based 2D/3D Perovskite Heterojunction with Funnel-Like Energy Band Alignment for Efficient Solar Cells (23.91%). Adv. Energy Mater. 2021, 11, 2102236 10.1002/aenm.202102236.

[ref75] Min H.; Lee D. Y.; Kim J.; Kim G.; Lee K. S.; Kim J.; Paik M. J.; Kim Y. K.; Kim K. S.; Kim M. G.; et al. Perovskite solar cells with atomically coherent interlayers on SnO_2_ electrodes. Nature 2021, 598, 444–450. 10.1038/s41586-021-03964-8. 34671136

[ref76] Zhang D.; Li H.; Riaz A.; Sharma A.; Liang W.; Wang Y.; Chen H.; Vora K.; Yan D.; Su Z.; et al. Unconventional direct synthesis of Ni_3_N/Ni with N-vacancies for efficient and stable hydrogen evolution. Energy Environ. Sci. 2022, 15, 185–195. 10.1039/D1EE02013G.

[ref77] Zhao L.; Li Q.; Hou C.-H.; Li S.; Yang X.; Wu J.; Zhang S.; Hu Q.; Wang Y.; Zhang Y.; et al. Chemical Polishing of Perovskite Surface Enhances Photovoltaic Performances. J. Am. Chem. Soc. 2022, 144, 1700–1708. 10.1021/jacs.1c10842. 35041406

[ref78] Haris M. P. U.; Kazim S.; Ahmad S. Microstrain and Urbach Energy Relaxation in FAPbI_3_-Based Solar Cells through Powder Engineering and Perfluoroalkyl Phosphate Ionic Liquid Additives. ACS Appl. Mater. Interfaces 2022, 14, 24546–24556. 10.1021/acsami.2c01960. 35583343

[ref79] Chao L.; Xia Y.; Duan X.; Wang Y.; Ran C.; Niu T.; Gu L.; Li D.; Hu J.; Gao X.; et al. Direct and stable α-phase formation via ionic liquid solvation for formamidinium-based perovskite solar cells. Joule 2022, 6, 2203–2217. 10.1016/j.joule.2022.07.008.

