# Supplementary material for: Photovoltaic Performance of FAPbI3 Perovskite Is Hampered by Intrinsic Quantum Confinement
Source: ACS Energy Lett. 2023 May 10;8(6):2543–51. doi: 10.1021/acsenergylett.3c00656 (PMC10262274; doi:10.1021/acsenergylett.3c00656)
Supplement: Supplementary file 1 — nz3c00656_si_001.pdf [file nz3c00656_si_001.pdf]

Supporting Information: Photovoltaic Performance of $FAPbI_3$ Perovskite is Hampered by Intrinsic Quantum Confinement

Karim A. Elmestekawy[1], Benjamin M. Gallant[1], Adam D. Wright[1], Philippe Holzhey[1], Nakita K. Noel[1], Michael B. Johnston[1], Henry J. Snaith[1], Laura M. Herz[1,2]

[1] Clarendon Laboratory, Department of Physics, University of Oxford, Parks Road, Oxford OX1 3PU, United Kingdom

[2] Institute for Advanced Study, Technical University of Munich, Lichtenbergstrasse 2a, D-85748 Garching, Germany

Email: laura.herz@physics.ox.ac.uk

1 Materials

Fluorine-doped tin oxide (FTO) coated glass substrates (8 or 15 $\Omega\,cm^{-2}$, AMG), z-cut quartz substrates (UQG Optics Ltd, 13 mm diameter), tin (IV) oxide (15 wt. % in H_2O colloidal dispersion, Alfa Aesar), lead(II) iodide (99.99%, trace metal basis, Tokyo Chemical Industries), aluminium oxide nanoparticles (20 wt. % in isopropyl alcohol, Sigma Aldrich), methylammonium iodide (>99.99%, Greatcell Solar Materials), formamidinium iodide (>99.99%, Greatcell Solar Materials), phenethylammonium iodide (>99%, Greatcell Solar Materials), spiro-OMeTAD (2,2',7,7'-Tetrakis(N,N-di-p-methoxyphenylamino)-9,9'-spirobifluoren, >99.5%, Luminescence Technology Corp.), FK209 Co(III) TFSI salt (tris(2-(1H-pyrazol-1-yl)-4-tert-butylpyridine)cobalt(III) tri[bis(trifluoromethane)sulfonimide], 98%, Sigma Aldrich), bis(trifluoromethylsulfonyl)amine lithium salt (99.95%, Sigma Aldrich), gold pellets (99.999%, Kurt J. Lesker Company).
n-butylamine (99.5%, Sigma Aldrich), n-butanol (anhydrous, 99.8%, Sigma Aldrich), 2-propanol (anhydrous, 99.5%, Sigma Aldrich), chlorobenzene (anhydrous, 99.8%, Sigma Aldrich), acetonitrile (anhydrous, 99.8%, Sigma Aldrich), 4-tert-butylpyridine (98%, Sigma Aldrich).

Prior to use, all FTO-coated and quartz substrates were scrubbed with an aqueous 2 vol% Decon 90 solution, rinsed with deionised water, sonicated in acetone and subsequently sonicated in 2-propanol before being dried in a stream of N_2 gas.
All other non-aqueous chemicals were stored in a N_2-filled glovebox before use and protected from exposure to light.

2 Fabrication

2.1 For material characterization:

A solution was prepared with 1:150 (vol:vol) aluminium oxide nanoparticles and anhydrous isopropyl alcohol. The solution was allowed to stir overnight prior to use. 80 μL of this solution was spin-coated dynamically on top of UV-ozone treated (15 minutes) z-cut quartz substrates, and annealed for 10 minutes at 100 °C. This process was carried out in ambient air. Subsequently a $FAPbI_3$ perovskite layer was deposited through either of the three methods described in the solar cell fabrication methods below.

2.2 For perovskite solar cell fabrication:

Fluorine-doped tin oxide (FTO) substrates (30 mm × 30 mm, 15 Ωcm^{-2}) were cleaned (scrubbed and ultrasonicated) with a solution of decon 90 (3 vol%) in deionised water, then rinsed and ultrasonicated sequentially with deionised water, acetone and isopropyl alcohol, then dried in a stream of N_2 gas.
A colloidal suspension of tin oxide nanoparticles (400 μL) was diluted with ultrapure water (2,600 μL). In an atmosphere containing minimum moisture (<5% relative humidity), 200 μL of this solution was placed statically on a UV-ozone treated (15 minutes) substrate coated with a fluorine-doped tin oxide layer (15 $\Omega\, cm^{-2}$), then spun at 4,000 rpm (with an initial acceleration of 1,000 rpm s^{-1}) for 30 seconds before being immediately annealed at 150°C for 30 minutes in the same environment. The substrates were allowed to cool, then immediately subjected to

a further 15 minutes of UV-ozone treatment before being immediately used in the following processing step.

One of three protocols was used for each of the $FAPbI_3$ perovskite thin films:

2.2.1 “DMF-DMSO” Method

Stoichiometric FAI and PbI_2 were dissolved in a 4:1 mixture of DMF:DMSO (DMF: N,N-dimethylformamide; DMSO: dimethylsulfoxide) at 1.4 M concentration. 150 μL of this solution was dispensed statically on top of the SnO_2 layer (for PSC devices) or Al_2O_3 nanoparticle-coated z-cut quartz substrate (for material characterisation) under an atmosphere of air controlled to 18-24% relative humidity. The substrate was spun at 6,000 rpm (with an initial acceleration of 12,000 rpm s^{-1}) for 35 seconds. At 10 seconds into this procedure, 800 μL of diethyl ether antisolvent was dispensed slowly onto the spinning substrate. The substrate was then heated in ambient air at 150°C for 10 minutes. This protocol is adapted from that described by Kim et al.[1].

2.2.2 “MACl route”

Stoichiometric FAI and PbI_2 were dissolved in a 4:1 mixture of DMF-DMSO at 1.8 M concentration, along with a 40 mol% excess of MACl. 150 μL of this solution was dispensed statically on top of the SnO_2 layer (for PSC devices) or Al_2O_3 nanoparticle-coated z-cut quartz substrate (for material characterisation) under an atmosphere of air controlled to 18-24% relative humidity. The substrate was spun at 6,000 rpm (with an initial acceleration of 12,000 rpm s^{-1}) for 35 seconds. At 10 seconds into this procedure, 800 μL of diethyl ether antisolvent was dispensed slowly onto the spinning substrate. The substrate was then heated in ambient air at 150°C for 10 minutes. This protocol is adapted from that described by Kim et al.[1], which reports minimal inclusion of any MA^+ or Cl^- ions in the resulting $FAPbI_3$ film. Additionally, similar fabrication methods reported in the literature have evidenced minuscule amounts of MA^+ ions (likely in the bulk of the film)[1,2] or Cl^- ions (likely at the lower interface in contact with the

substrate)[3,4] being incorporated. Such trace inclusions are highly unlikely to have any effect on the structure and electronic bandstructure, as apparent from absorption onsets reported for across the three fabrication methods.

2.2.3 "Sequential deposition"

Lead iodide (1.266 mmol, 583.5 mg) and methylammonium iodide (1.139 mmol, 181.1 mg) were mixed with n-butylamine (1.899 mmol, 187.6 μL) and the ether solvent (1,500 μL) and agitated until all solids were fully dissolved, in a modified version of the process reported by Noel et al.[5]. 50 μL of this solution was spin-coated dynamically on top of the SnO_2 layer (in PSC devices) or Al_2O_3 nanoparticle-coated z-cut quartz substrate (for material characterisation) at 2,500 rpm for 45 seconds. The substrates were immediately annealed at 70°C for 10 minutes. After cooling, the substrates were coated with 350 μL of a 0.1 M solution of formamidinium iodide (0.500 mmol, 86.0 mg) dissolved in n-butanol (5,000 μL). After 45 seconds of static soaking, the substrates were spun at 4,000 rpm (with an initial acceleration of 1,000 rpm s^{-1}) for 45 seconds, then immediately annealed for 10 minutes at 70°C, followed by 30 minutes at 180°C.

In all cases, the substrates were allowed to cool, then a 20 mM solution of phenethylammonium iodide (0.200 mmol, 49.8 mg) in 2-propanol (10,000 μL) was spin-coated dynamically on top at 5,000 rpm for 45 seconds, in a N_2-containing glovebox.

A solution of spiro-OMeTAD (0.070 mmol, 85.8 mg) dissolved in chlorobenzene (1,000 μL) and doped with 8.4 μL of a 0.250 M solution of tris(2-(1H-pyrazol-1-yl)-4-tert-butylpyridine)cobalt(III) tri[bis(trifluoromethane)sulfonimide] in acetonitrile, 19.4 μL of a 1.8 M solution of bis(trifluoromethylsulfonyl)amine lithium salt in acetonitrile, and tert-butyl pyridine (0.231 mmol, 38.0 μL) was spin-coated on the PEAI-passivated substrates dynamically at 2,500 rpm for 30 seconds, in a N_2-containing glovebox.

Finally, 80 nm of gold was evaporated on top of the substrates at an initial rate of 0.1 As^{-1} at a pressure $<2\times10^{-6}$ torr.

3 X-ray diffraction (XRD)

XRD patterns were measured in air using a Panalytical X'pert powder diffractometer with a copper X-ray source (Cu-Kα X-rays with a wavelength of 1.5418 Å).

The XRD patterns were then corrected for tilt by shifting the 2θ-axis, such that the z-cut quartz reference peaks at $2\theta = 16.433°$ were all aligned for the films on quartz substrates.

In this study, the thicknesses of the films were optimised for each fabrication method to yield the best performing photovoltaic devices with the highest crystallinity and most favorable optoelectronic behavior. The examined films are therefore of different thicknesses (as measured from cross-sectional SEM images shown in Section 4). This difference in thickness will, to an extent, affect the absolute intensities of the XRD peaks. We therefore focus our attention on the relative intensities of the main perovskite peaks and those of crystallographic defect phases present in the system. As expected, the $FAPbI_3$ films fabricated using the MACl additive-based route and sequential deposition method exhibit superior perovskite crystallinity, improved crystallographic purity, better perovskite conversion, and lower relative intensity peaks arising from crystallographic impurities. The significantly lower XRD peak intensity for PbI_2 precursor remnants relative to the main perovskite peak signifies better perovskite conversion for the $FAPbI_3$ films fabricated from the MACl route and sequential deposition method (see Figure 1a). The presence of XRD peaks associated with the different hexagonal polymorphs at 11.5°[6], and 11.8°[6,7] for the $FAPbI_3$ films fabricated via the neat DMF-DMSO route, along with their substantially larger relative intensity with respect to the main perovskite peaks demonstrate the film's relatively poor crystallographic purity and highlight the stronger presence of the δ-phase along with a plethora of other hexagonal polymorphs[6] in such films (see Figure 1a). Such poor crystalline quality existing for the films fabricated via the neat DMF-DMSO route is additionally evident from the significantly higher intensity of the quartz substrate peak at $2\theta = 16.22°$ relative to the (100) and (200) main perovskite peaks in the full XRD patterns in Supporting Figure 1, which is evidence for poor perovskite conversion even for a 340 nm thick film.

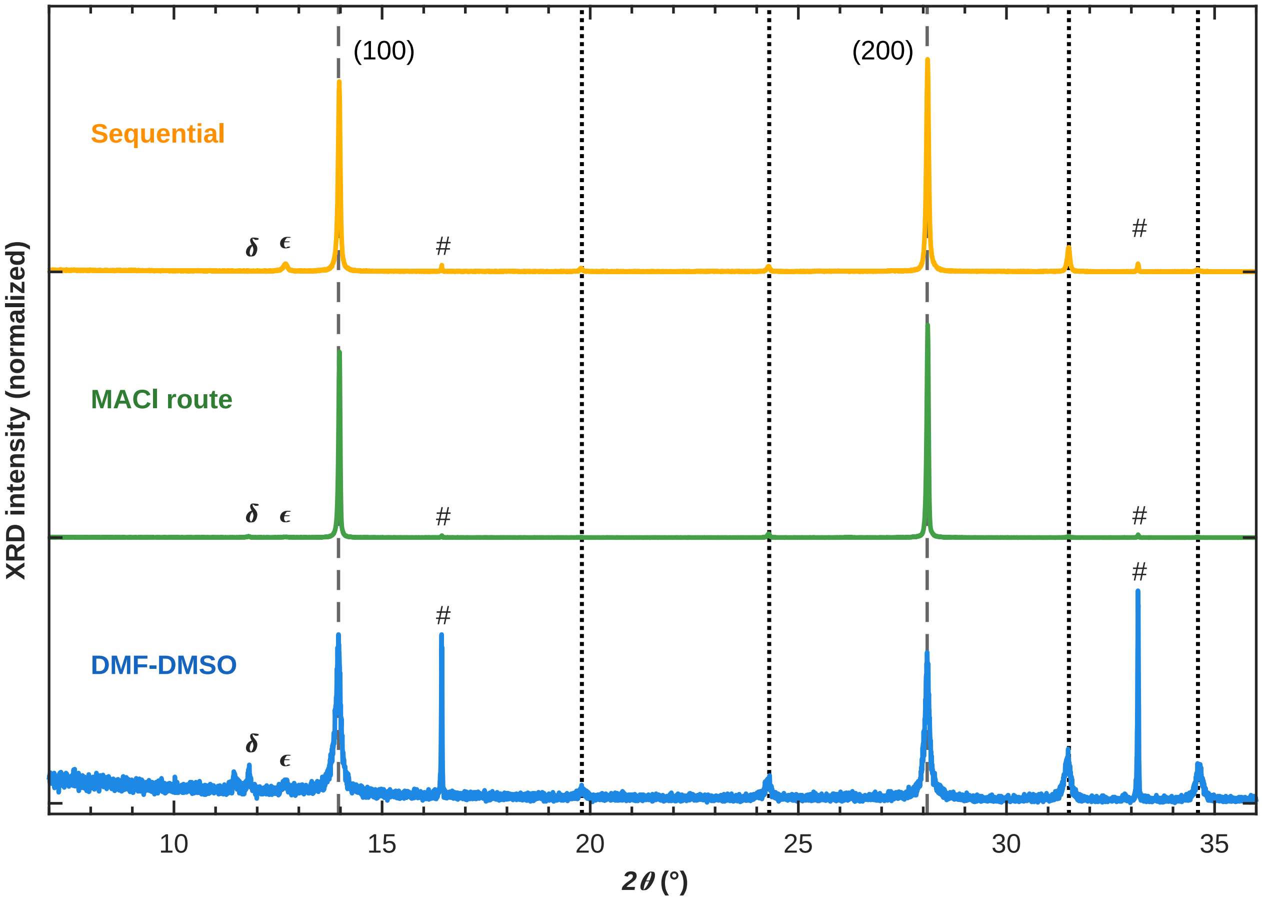

Supporting Figure 1: Normalized X-ray diffraction (XRD) pattern for $FAPbI_3$ thin films fabricated via DMF-DMSO, MACl route and sequential deposition method. The quartz substrate peak is indexed with #. The main pseudo-cubic α-$FAPbI_3$ peaks ((100) and (200)) are represented by the vertical black dashed lines at the experimentally measured values of 14° and 28.1° respectively. The literature values[7] of the less prominent cubic α-$FAPbI_3$ peaks are indicated by vertical black dotted lines, at 2θ values of $19.8°, 24.3°, 31.5°$, and 34.6°. The PbI_2 peak at 12.7° is indexed with ϵ[8]. The most prominent XRD peak for the non-perovskite δ-$FAPbI_3$ phase appears at 11.8°,[6,7] and is indexed accordingly.

The extracted peak positions and full widths at half maximum (FWHM) of the pseudo-cubic (100) perovskite XRD peak of α-$FAPbI_3$ were obtained by using the extracted fitting parameters from pseudo-Voigt fits.

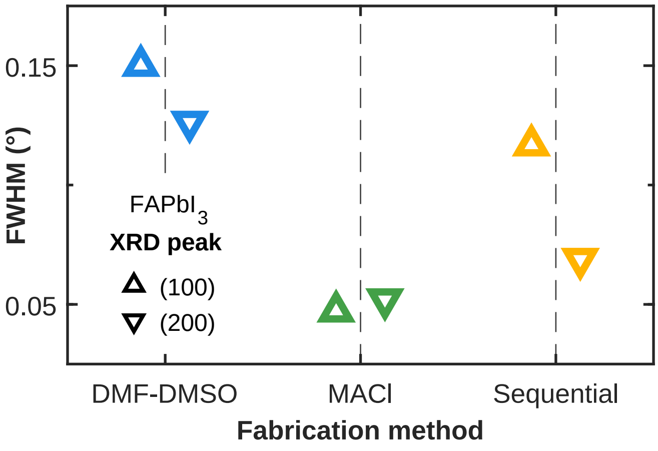

Supporting Figure 2: Full widths at half maximum (FWHM) of the prominent (100) α-$FAPbI_3$ XRD perovskite peak for $FAPbI_3$ thin films fabricated via DMF-DMSO, MACl route and sequential deposition method. Extracted FWHM of the two most prominent XRD peaks (100), and (200) for the investigated films.

3.1 Synchrotron X-ray data

Synchrotron X-ray scattering data was acquired at the Stanford Synchrotron Radiation Lightsource (SSRL) at beamline 11-3. The samples were measured at an incident angle of 3° and an incident X-ray energy of 12.7 keV. A Rayonix MX225 2D detector was used for data collection and a LaB_6 standard was used to calibrate the sample to detector distance. The data were integrated over the full azimuthal range using GSAS-II. Peaks were fitted with a Voigt function.

a b c

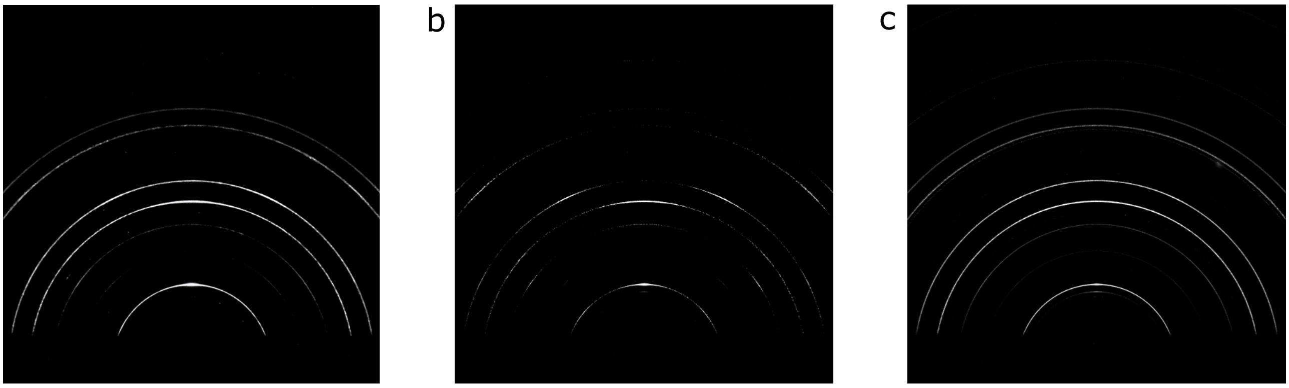

Supporting Figure 3: Grazing-incidence wide angle X-ray spectroscopy of $FAPbI_3$ thin films fabricated via a. DMF-DMSO, b. MACl route and c. sequential deposition method.

4 SEM

A FEI Quanta 600 FEG Environmental Scanning Electron Microscope (ESEM) was employed to investigate perovskite layer morphology. Accelerating voltages between 4-15 kV were employed for various analyses. For cross-sectional SEM images the substrates were partially scored post-deposition, then snapped along the score line. Images were acquired from snapped regions that had not been directly scored.

The top-down SEM images highlight the morphological changes occurring in the absorber layer when the fabrication protocol is varied.

The cross-sectional SEM images provide accurate measurements of the thicknesses of the films made from the different fabrications methods. They additionally highlight the substantially enlarged and monolithic grain structure for the $FAPbI_3$ thin films fabricated via the MACl route and sequential deposition method, compared to the films made via the neat DMF-DMSO route sufferring from intragrain structures, similar to previous reports in the literature[1,9].

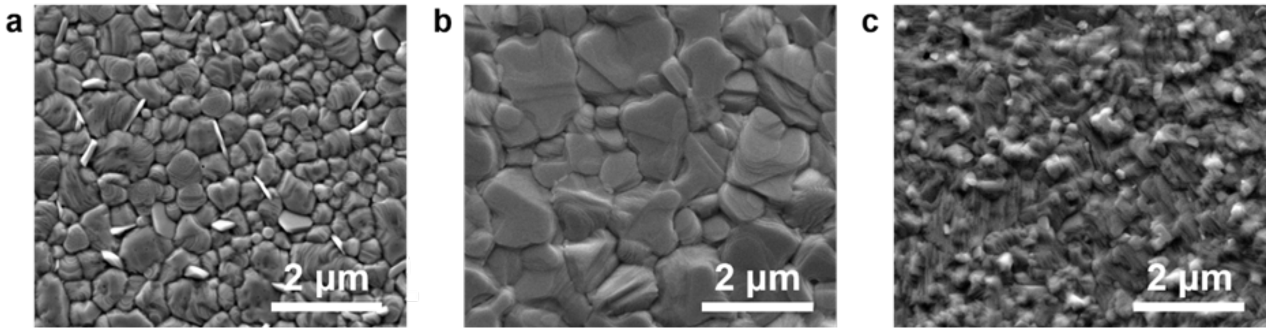

Supporting Figure 4: Top-down SEM images of photovoltaic devices incorporating the differently-fabricated $FAPbI_3$ films. **a**. $FAPbI_3$ film made via neat DMF-DMSO route, **b**. $FAPbI_3$ film fabricated through the MACl route, and **c**. $FAPbI_3$ made via the sequential deposition route.

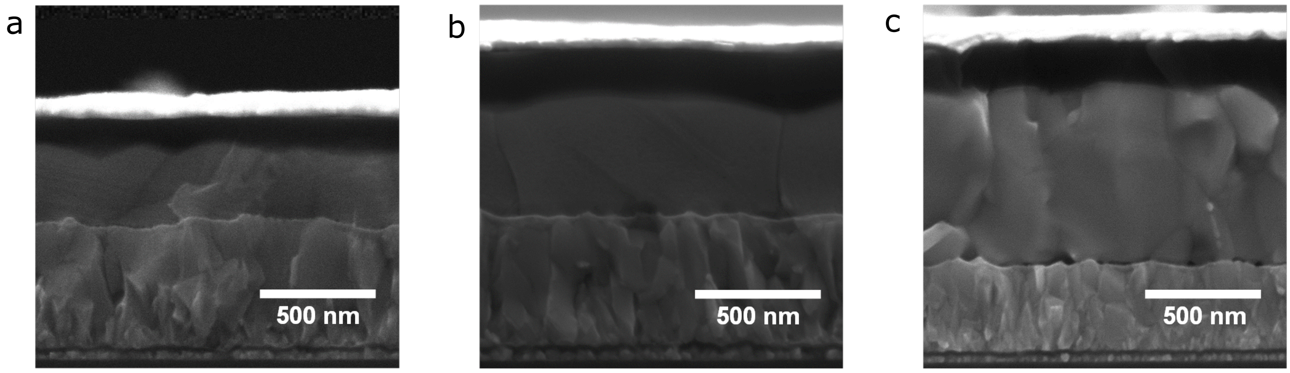

Supporting Figure 5: Cross-sectional SEM images of photovoltaic devices incorporating the differently-fabricated $FAPbI_3$ films. **a**. $FAPbI_3$ film made via neat DMF-DMSO route, with an optimal thickness of 340 nm, **b**. $FAPbI_3$ film fabricated through the MACl route, with an optimal thickness of 440 nm, and **c**. $FAPbI_3$ made via the sequential deposition route, with an optimal thickness of 770 nm. This device stack comprises FTO, SnO_2 nanoparticles, perovskite layer with a PEAI surface treatment, Spiro OMeTAD, Au electrodes.

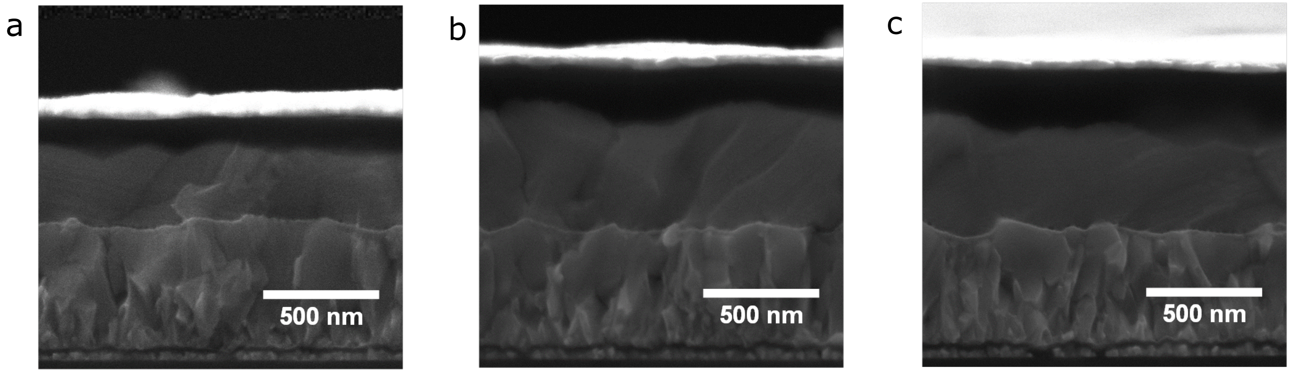

Supporting Figure 6: Cross-sectional SEM images of the devices incorporating $FAPbI_3$ films made via the neat DMF-DMSO route, with different molar concentrations of FAI. **a**. 1.4 M (resulting in $FAPbI_3$ thickness of 340 nm) **b**. 1.6 M (resulting in $FAPbI_3$ thickness of 450 nm), and **c**. 1.8 M (resulting in $FAPbI_3$ thickness of 440 nm). This device stack comprises FTO, SnO_2 nanoparticles, perovskite layer with a PEAI surface treatment, Spiro OMeTAD, Au electrodes.

Employing different concentration of the $FAPbI_3$ precursor solution for the films made via the neat DMF-DMSO method resulted in films of different thicknesses. The film thickness increase as the precursor concentration increases from 1.4 M to 1.6 M, followed by a noticeable decrease when the precursor concentration further to 1.8 M, which could be a result of incomplete conversion of $FAPbI_3$ to the perovskite phase and the expected fast nucleation associated with higher precursor and colloid concentrations. By contrast, $FAPbI_3$ fabricated via the MACl route gives optimal performance when a 1.8 M precursor solution is employed.

5 Absorption measurements

Reflectance (R) and transmittance (T) spectra were measured using a Fourier transform infrared (FTIR) spectrometer (Bruker Vertex 80v), configured with a tungsten halogen lamp illumination source, a CaF_2 beamsplitter and a silicon detector. The absorption coefficient (α) spectrum of each thin-film sample is then obtained using the relationship

$$\alpha = -\frac{1}{d} \cdot \log_{10}\left(\frac{T}{1-R}\right)$$

where d is the film thickness.

The samples were all mounted in a gas-exchange helium cryostat (Oxford Instruments, OptistatCF2) for the room-temperature measurements (where the outer vacuum chamber was pumped down to low pressures ($< 5 \times 10^{-5}$ mbar) and for the temperature-dependent study, where the temperature was varied between 4K and 295K in either 5K or 10K increments.

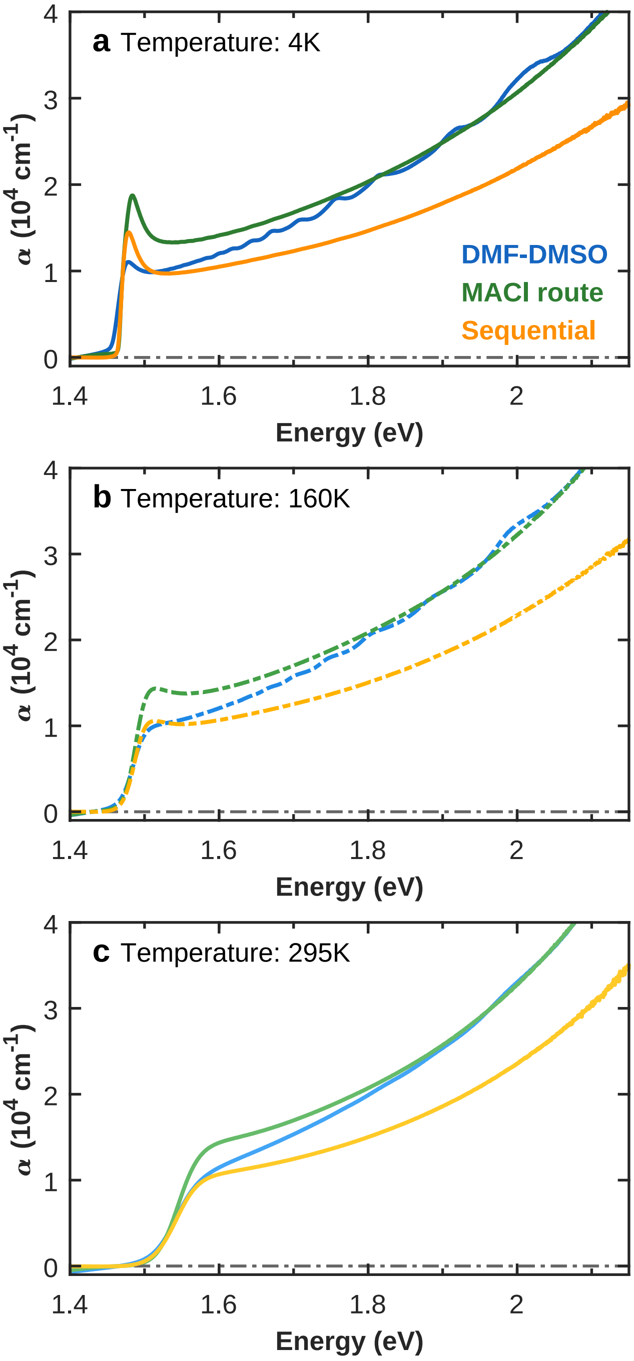

Supporting Figure 7: Absorption coefficient spectra of $FAPbI_3$ thin films fabricated via DMF-DMSO, MACl route and sequential deposition method at a. 4 K, b. 160 K, and c. 295 K.

5.1 Elliott fitting

The onset in the absorption coefficient spectrum is fitted using Elliott's model that describes the absorption of a semiconductor near its band edge.[10]

The theory expresses the energy-dependent absorption as:

$$\alpha(E) = \alpha_{\mathrm{X}}(E) + \alpha_{\mathrm{C}}(E)$$

The bound exciton contribution α_{X} has the form:

$$\alpha_{\mathrm{X}}(E) = \frac{b_0}{E} \sum_{n=1}^{\infty} \frac{4\pi E_{\mathrm{b}}^{3/2}}{n^3} \delta\left(E - \left[E_{\mathrm{g}} - \frac{E_{\mathrm{b}}}{n^2}\right]\right)$$

where b_0 is a constant of proportionality that incorporates the electric dipole transition matrix element between the valence and conduction band. α_{X} is formed of the weighted sum of contributions from the exciton states with positive integer quantum number n and energies $E_{\mathrm{g}} - \frac{E_{\mathrm{b}}}{n^2}$, where E_{g} is the band gap energy and E_{b} is the exciton binding energy.
Here, the contribution from the electron-hole continuum states $\alpha_{\mathrm{C}}(E)$ has the form

$$\alpha_{\mathrm{C}}(E) = \frac{b_0}{E} \left[\frac{2\pi\sqrt{\frac{E_{\mathrm{b}}}{E-E_{\mathrm{g}}}}}{1 - \exp(-2\pi\sqrt{\frac{E_{\mathrm{b}}}{E-E_{\mathrm{g}}}})} \right] c_0^{-1} \mathrm{JDoS}(E)$$

where the joint density of states is given by $\mathrm{JDoS}(E) = c_0\sqrt{E - E_{\mathrm{g}}}$ for $E > E_{\mathrm{g}}$ and 0 otherwise, and the joint density of states constant $c_0 = \frac{1}{(2\pi)^2}\left(\frac{2\mu}{\hbar^2}\right)^{3/2} \times\ 2$, where μ is the reduced effective mass of the electron-hole system. The term in square brackets is the Coloumbic enhancement factor, which increases the absorption from the continuum states above the square-root form of the JDoS for a direct-gap semiconductor as a result of the Coloumbic attraction between the unbound electrons and holes.
The linear combination of the contributions from the excitonic and continuum states, $\alpha(E)$, is then convolved with a broadening normal distribution function - representing the homoge-

neous broadening caused by electron-phonon coupling, disorder and local fluctuations of the stoichiometry of the material. The broadening is mathematically denoted by $g(E) = \mathcal{N}(0, \sigma_\mathrm{T}^2)$.

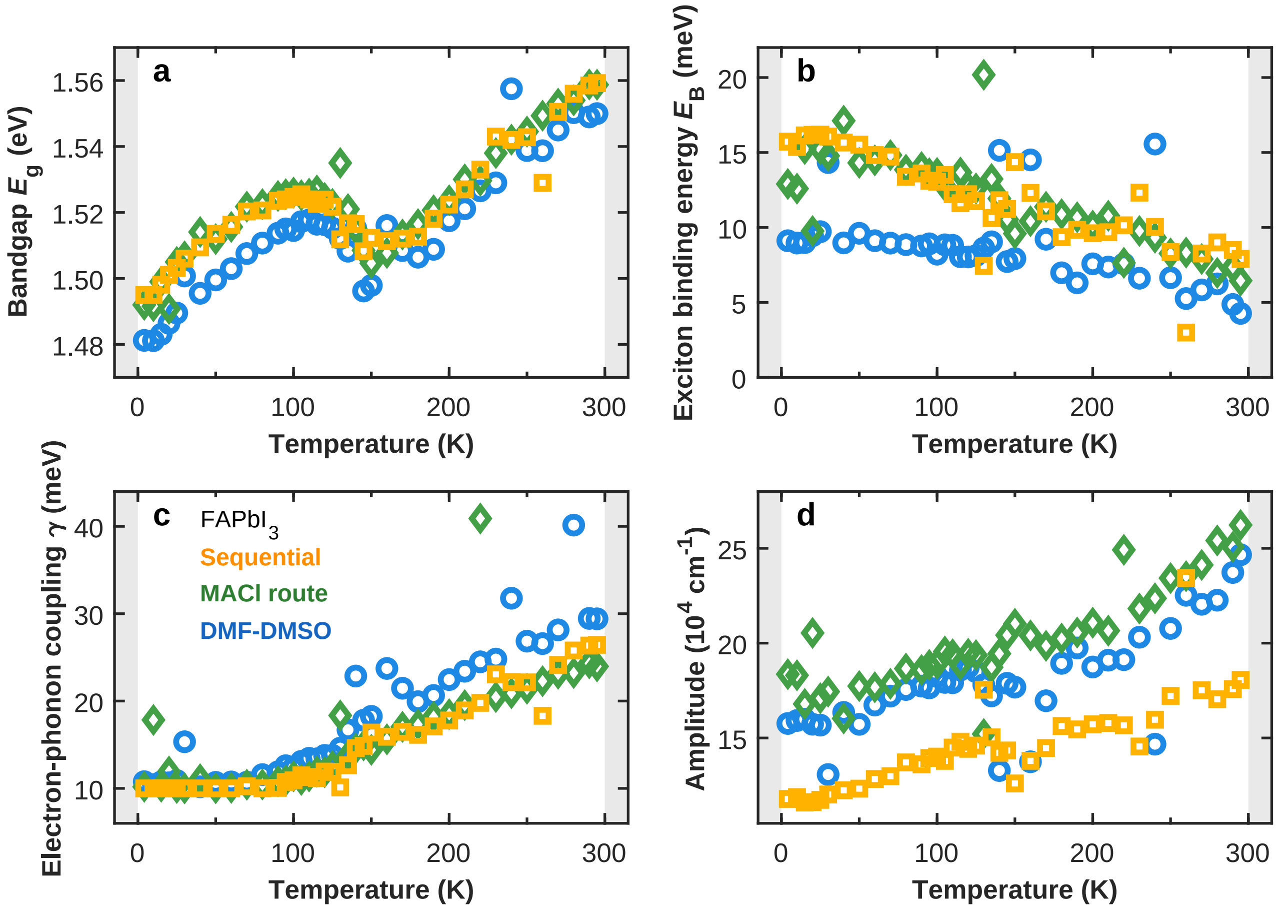

Supporting Figure 8: Extracted fitted parameters from Elliott fitting of the absorption onset of stoichiometric $FAPbI_3$ thin films fabricated via DMF-DMSO, MACl route and sequential deposition method. The absorption coefficient spectra were fitted with 4 free parameters: E_g representing the optical bandgap, E_b representing the exciton binding energy, γ representing the broadening arising from energetic disorder within the film, and Amp representing the amplitude of the spectrum.

5.2 Baseline fitting

Elliott fitting is only applicable to the absorption onset near the band edge, therefore to decouple the oscillatory features from the standard absorption coefficient spectrum at energies higher than the bandgap, we used a phenomenological fit to extract the peaks and quantify their behaviour, as described in our previous works[11,12].

A smoothing algorithm is applied to the peaked features of the absorption spectrum and subsequently subtracted from the absorption spectrum to result in purely the contributions from the peak features, whose minima correspond to the troughs observed in the experimental data. Interpolating a cubic spline between the experimental data at the energies of the minima, resulted in the baseline fit we used to decouple the peak features from the underlying absorption coefficient data resembling the bulk material.

It should be noted that this procedure also eliminates any possible additional density-of-state contributions associated with the quantum confinement.

5.3 Temperature dependence of the absorbance spectra

A reduction in temperature is expected to increase the prominence of the studied absorption features and by association the strength of the electronic quantum confinement. This experienced increase in confinement strength may arise from the lattice contraction and subsequent reduction in confinement length scale, and a decrease in electron-phonon coupling and thermal fluctuations[11,12]. In addition, the increased prominence may derive from a growth in the material volume experiencing confinement as the formation of the potentially-confining δ-phase becomes more prominent at lower temperatures[11–13].

5.4 Definitions

In our quantitative analysis of the quantum confinement and behaviour of the peaks, we utilise the confinement energy and spectral area under the peaks. We define the confinement energy associated with a given peak as the peak energy position of the decoupled peak minus the Elliott-extracted band gap of the material. Meanwhile, the spectral area under the peaks is defined as the spectral area between the experimental absorption coefficient data and baseline fit, stated as a percentage of the spectral area under our experimental data (integrated between the first and last detectable minima of the oscillatory peak features).

6 Photoluminescence

A 398 nm diode laser (PicoHarp, LDH-D-C-405M) was used to photo-excite the samples, on a continuous wave setting at an intensity of 67.3 mW/cm^2. The resultant PL was collected and coupled into a grating spectrometer (Princeton Instruments, SP-2558), which directed the spectrally dispersed PL onto a silicon iCCD (intensified charge coupled device, PI-MAX4, Princeton Instruments). The samples were mounted in a vacuum cell under low pressure ($\sim 10^{-2}$ mbar).

The extracted optical bandgap from the maximum intensity of the room-temperature PL spectra of the $FAPbI_3$ films is shown in Supporting Figure 9, and is further proof that the $FAPbI_3$ thin films fabricated via DMF-DMSO, MACl route and sequential deposition method are of essentially the same composition.

We note that similar low-temperature PL spectra of a $FAPbI_3$ film exhibiting strong quantum confinement have previously been reported,[12] with steady-state PL peaks falling at similar energies to those of corresponding peaks in the absorption spectra, and time-resolved PL showing differences between bulk and confined phases.[12]

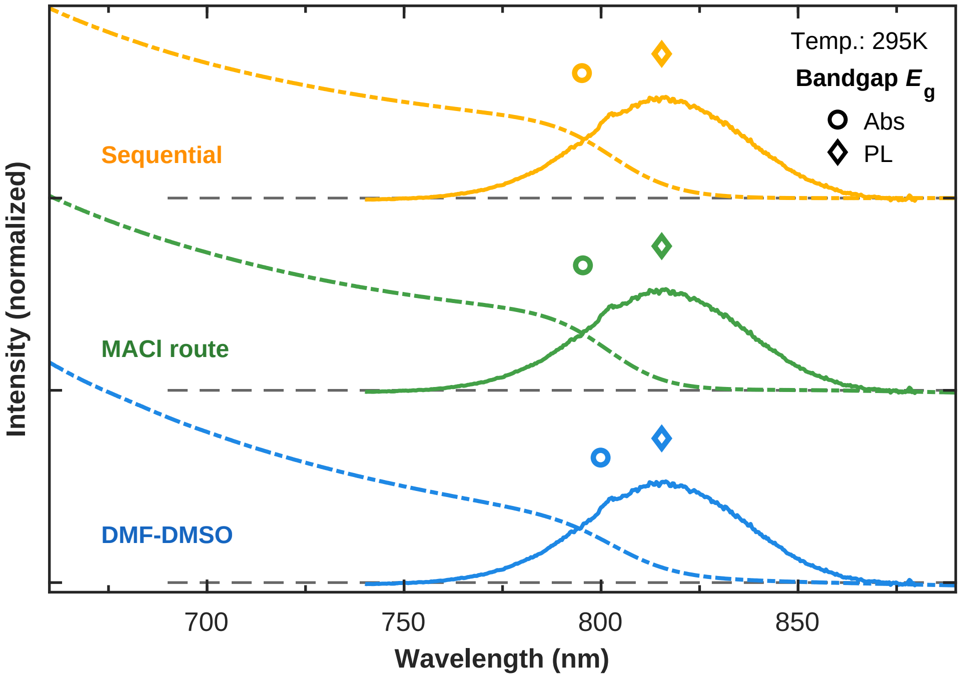

Supporting Figure 9: Room temperature photoluminescence and absorption coefficient spectra of stoichiometric $FAPbI_3$ films fabricated via DMF-DMSO, MACl route and sequential deposition method. The small peak feature near 800 nm originates from the second-order diffraction of the scattered laser excitation centred at 398 nm.

7 Photovoltaic performance

Current-voltage (J–V) and maximum power point (MPP) measurements were carried out using a 2400 series source meter by Keithley Instruments in ambient air both under light (simulated AM 1.5 irradiance generated by a Wavelabs SINUS-220 simulator) and in the dark. The active area of the solar cell was masked with a black-anodised metal aperture to 0.25 cm^2, within a light-tight holder. Device areas are determined based on the aperture area of a mask that covers the solar cell during measurement. These masks have been measured precisely using optical microscopy (area is correct ± 0.5%).[14] The "forward" J–V scans were measured from forward bias to short-circuit and the "backward" scans were from short-circuit to forward bias, both at a scan rate of 245 mVs^{-1}. Active MPP tracking measurements using a gradient descent algorithm were performed for at least 30 s to obtain the steady-state power conversion efficiency. Active MPP tracking measurements (η_{mpp}) using a gradient descent algorithm were performed for at least 30 s to obtain the steady-state power conversion efficiency. Steady-state open-circuit voltage (V_{OC}) measurements were performed by illuminating the PSCs and extracting no charge carriers ($J = 0\,\mathrm{mAcm^{-2}}$) and measuring the potential difference across the cell until it no longer evolved over time (10-250 s). Subsequently for each PSC, a steady-state short-circuit current (J_{SC}) measurement was made by illuminating the PSCs and holding the applied bias at 0 V and measuring the current density output over time until it no longer evolved over time (5-20 s). Some cells exhibited improvement over multiple measurements, in which case the peak performance was reported. This typically took two to five J–V scan plus MPP tracking iterations for the highest efficiency cells, in a measurement time of around 2–5 minutes. The intensity of the solar simulator was set periodically such that the short-circuit current density from a KG3-filtered Si reference photodiode (Fraunhofer ISE) matched its 1-sun certified value. A local measurement of the intensity before each batch of solar cell measurements were performed by integrating the spectrum obtained from the solar simulator's internal spectrometer. By taking the ratio of this internal intensity measurement to one obtained at the time of calibration we determined the equivalent irradiance at the time of measurement.

For the data presented in this publication, this gave values ranging from 0.990-1.005 suns equivalent, which have been applied to the calculation of power conversion efficiencies for each individual measurement. The spectral mismatch factor was estimated to be 1.022 according to a previously reported method.[14] This has also been applied to calculate power conversion efficiencies. We estimate the systematic error of this setup to be on the order of ± 5% (relative).

EQE spectra of two sample solar cells incorporating $FAPbI_3$ films produced through the MACl route and the sequential deposition approach are presented in Supporting Figure 13 together with their corresponding J-V characteristics. We note that the integrated J_{SC} values for both PSCs presented are somewhat lower than those exacted from the J-V characteristics of the same cell or found from a steady-state J_{SC} measurement. A similar disparity has been reported by a number of research groups, and was recently described and summarised in detail in a Viewpoint from Saliba and Etgar,[15] and observed by Lin et al.[16] and Schutt et al.[17]. In these examples the difference between the integrated J_{SC} measured via EQE and the steady-state J_{SC} extracted from J-V characteristics is of similar magnitude to that observed in this study. We further calibrated our solar simulator, by carrying out a stabilised J_{SC} measurement (whereby the bias across the solar cell is held at 0 V until the extracted current stabilises) to confirm that current densities extracted from J-V characteristics do not over- or underestimate J_{SC}, which is of particular concern for PSCs in the n-i-p configuration given that these devices are prone to hysteretic behaviour. Accordingly, the steady-state J_{SC} values presented in our manuscript are precise to an acceptable degree of error.

Throughout the photovoltaic comparison of these films, we are assuming the films are compositionally alike and similar enough that the steady-state J_{SC} is an accurate reflection of the intrinsic efficiency with which the photoexcited charge carriers move through the film. Some small differences between these films may however exist which may slightly affect their PV properties. The energy landscape of each film and how well their conduction band minimum (CBM) and valence band maximum (VBM) match those of the transport layers may slightly

vary across the different fabrication methods because of the difference in lattice distortions present in the crystal. The tilting of the perovskite octahedra or the slight change in the lattice parameter caused by a contraction or an expansion as a result of extra stress or relief in the system can cause a change in the orbital overlap of the Pb and I ions which would slightly change the positions of the CBM and VBM[18,19], changing the energetic alignment with the transport layers, and yet still resulting in similar bandgaps. However, a better-matched perovskite band alignment with the transport layers would mainly result in an improvement in the steady-state V_{OC}, maintaining the validity of our method of using the steady-state J_{SC} as an accurate gauge of how unobstructed the photocurrent is.

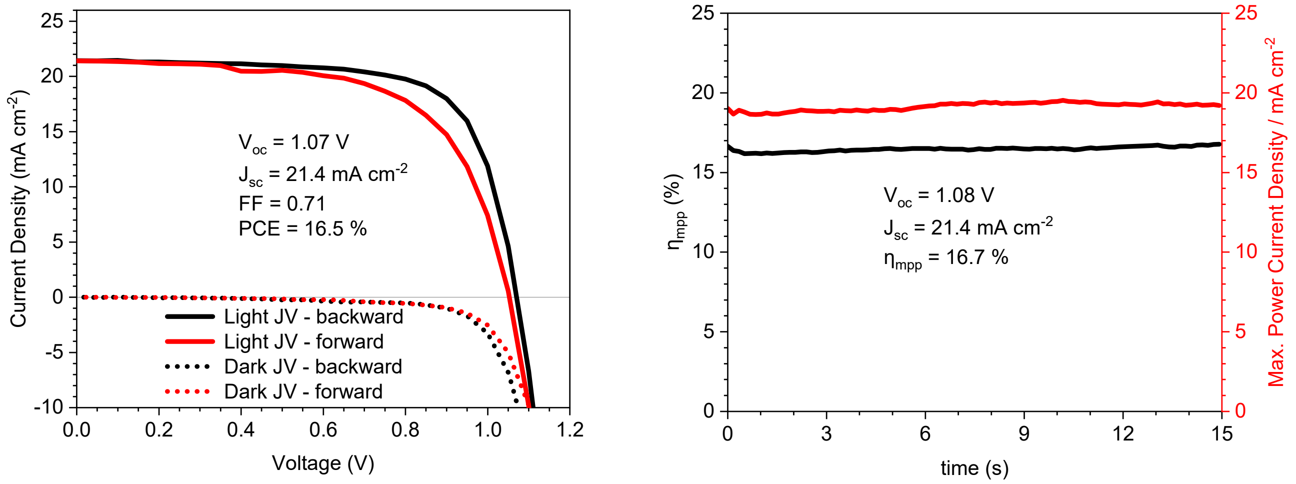

Supporting Figure 10: $J-V$ characteristics and maximum power tracked power output plots of the champion cell incorporating an $FAPbI_3$ layer fabricated via the neat DMF-DMSO route.

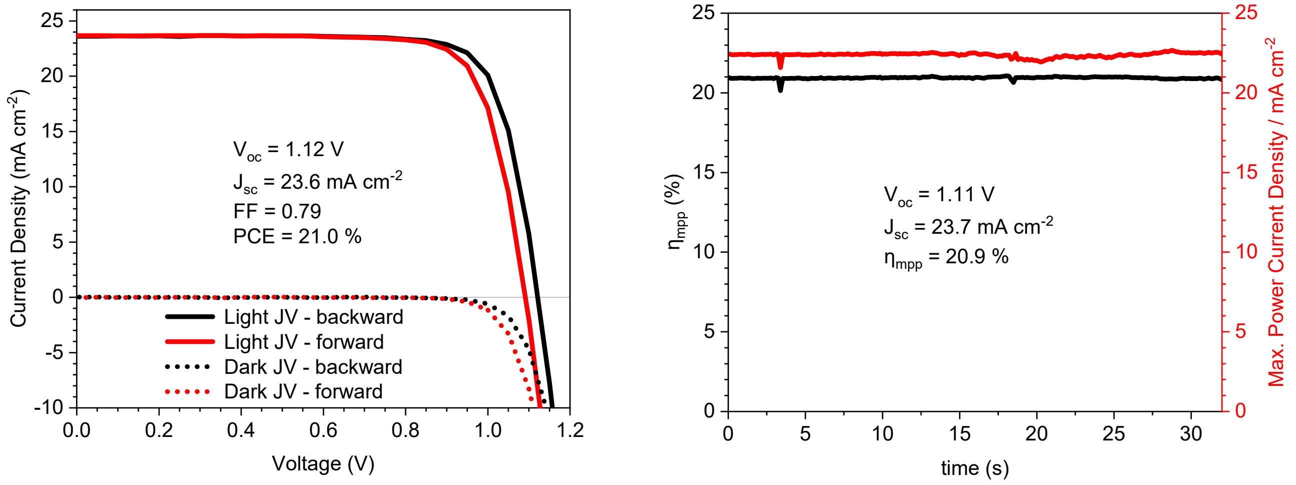

Supporting Figure 11: $J - V$ characteristics and maximum power tracked power output plots of the champion cell incorporating an $FAPbI_3$ layer fabricated via the MACl route.

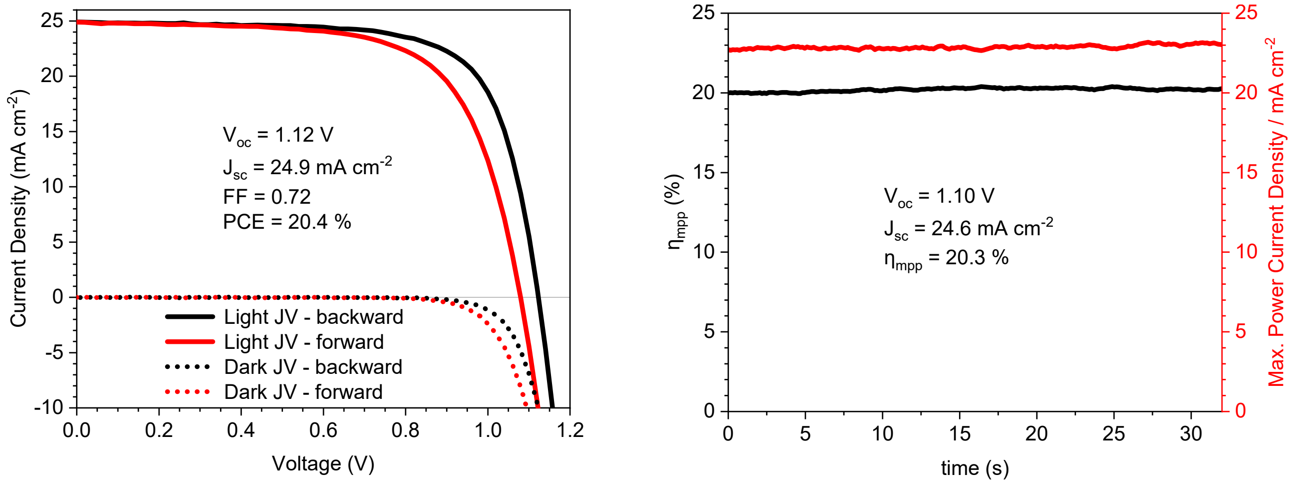

Supporting Figure 12: $J - V$ characteristics and maximum power tracked power output plots of the champion cell incorporating an $FAPbI_3$ layer fabricated via the sequential deposition route.

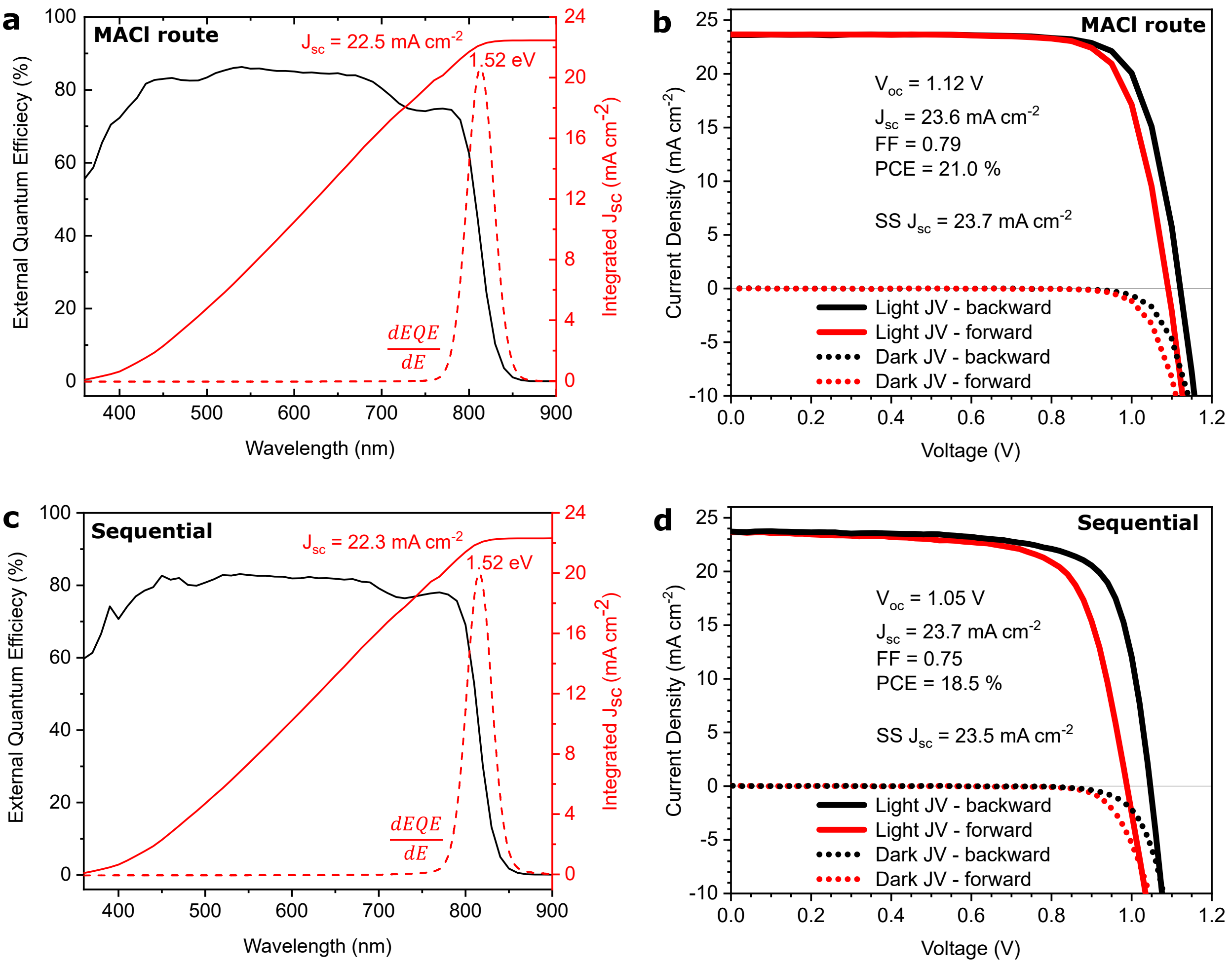

Supporting Figure 13: "EQE spectra and $J-V$ characteristics of two sample solar cells incorporating a $FAPbI_3$ layer fabricated either via the (a,b) MACl route or (c, d) the sequential deposition route. We note that these devices represent randomly selected examples rather than champion cells from within the batch.

To investigate in more detail the major increase in steady-state J_{SC} for solar cells incorporating $FAPbI_3$ layers fabricated through the MACl route and sequential deposition method, compared to those made via the neat DMF-DMSO route, we carried out a comparison of photovoltaic parameters for films made via the neat DMF-DMSO route, but with different molar concentrations of FAI, resulting in films with different thicknesses. As shown in Section 4 in the SI, the resulting thickness values of the perovskite films are 340 nm, 450 nm, and 440 nm for 1.4 M, 1.6 M, and 1.8 M FAI precursor solutions respectively. As can be seen in Supporting Figure 14, devices with thicker $FAPbI_3$ films perform substantially worse when compared to the 1.4 M-FAI $FAPbI_3$ film with 340 nm thickness (which represented the value for which the power conversion efficiency was optimised) with the V_{OC} and η_{mpp} for the thicker devices representing the main culprits behind the worsened PV performance. These results could reflect a more defective perovskite-transport layer interface, possibly caused by an increased surface roughness, or too rapid a crystallization process resulting from the higher concentration $FAPbI_3$ precursor solution used, which naturally supersaturates earlier in the deposition process.

More intriguingly, the steady-state J_{SC} values have a different dependence on the precursor concentration and film thickness than V_{OC} values. As discussed in Section 4 in the SI, the thickness of the $FAPbI_3$ films are 340 nm, 450 nm, and 440 nm for the 1.4 M, 1.6 M, and 1.8 M precursor solutions respectively, and the steady-state J_{SC} seems to generally follow the same variation in thickness. This suggests that these devices are, to a small extent, limited by the thickness of the photoabsorber layer. However, the comparison between the 440 nm-thick $FAPbI_3$ film made from the neat DMF-DMSO route (shown in Supporting Figure 14), and the equally-thick film made via the MACl route (Figure 2) sheds more light on the main factor limiting the charge-carrier extraction. Even with the increase in stabilsied J_{SC} owing to the use of a thicker $FAPbI_3$ film from the neat DMF-DMSO method, its value still lags behind that obtained for the film made from the MACl route by ca. $2\,mAcm^{-2}$.

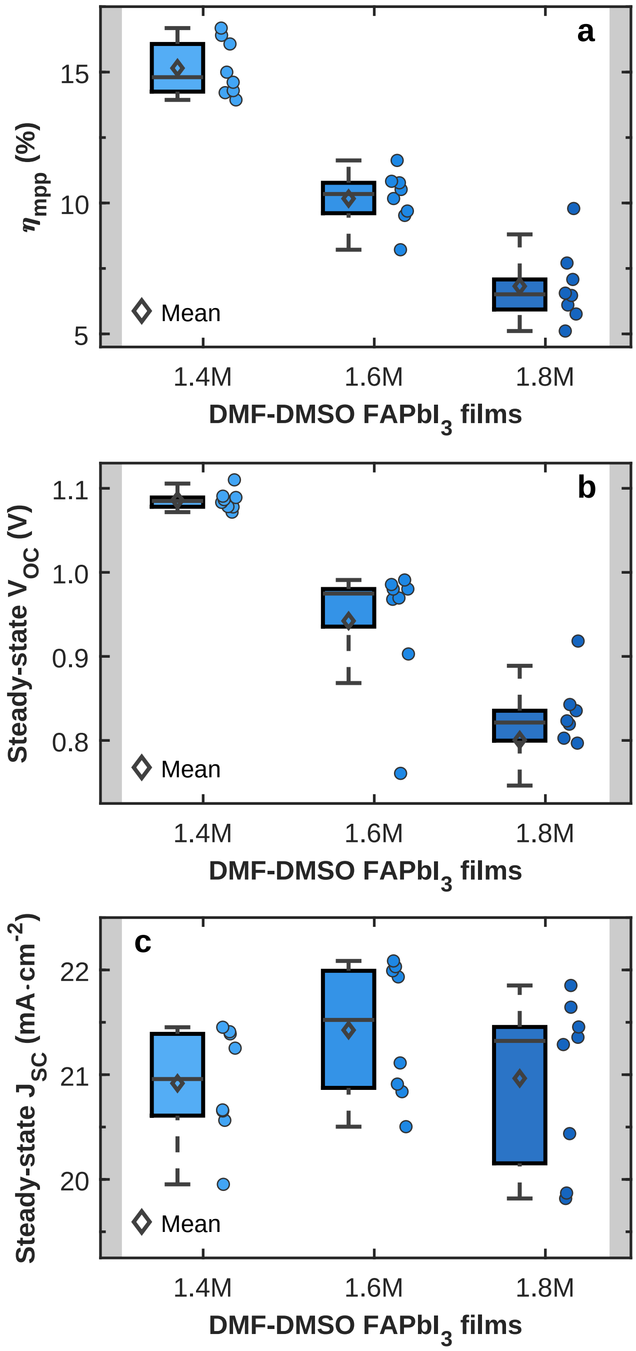

Supporting Figure 14: Performance of photovoltaic devices incorporating $FAPbI_3$ absorber layers made from the neat DMF-DMSO route using different FAI precursor molar concentrations that resulted in different film thicknesses. Box and scatter plots showing the photovoltaic device parameters obtained from $J - V$ curves for $FAPbI_3$ films made from the neat DMF-DMSO route using different FAI precursor molar concentrations that resulted in different film thicknesses; **a**. Maximum power point tracked power conversion efficiency, **b**. Steady-state V_{OC}, and **c**. Steady-state J_{SC}. The scatter points represent the different pixels investigated.

8 meta-analysis of literature reports

For our meta-study of literature reports covering photovoltaic applications with $FAPbI_3$ as absorber layer, we searched for relevant publications as follows:

- For reports with publication dates up to and until the 31st of December 2019, we utilised the open-access Perovskite Database Project,[20] which reports photovoltaic devices incorporating perovskites as their photo-absorber layer and the performance for their corresponding PV devices. This dynamic database is thorough and comprehensive up to the chosen date of the 31st of December 2019, with some additional sporadic entries beyond that from authors choosing to upload PV performance data for their devices after the release of the database.

- For reports from the $1st$ of January 2020, we searched the "Web of Science"[21] global citation database by entering "Formamidinium" as the search term in the abstract or title of all published scientific reports, as a starting point to obtain a similarly comprehensive overview of the field working on fabricating solar cells incorporating $FAPbI_3$.

Any publications not reporting PV performance data for solar cells incorporating $FAPbI_3$ films were excluded from our meta-study. For publications with such data available, the typical PCE values recorded in this study were either the averaged PCE values determined across a batch of many solar cell devices, or alternatively the champion PCE value of the solar cell if the averaged value is not reported, both during a reverse scan. We note that the field has evolved since the earliest publications we included (i.e. from December 2013 onwards) towards improved device reporting standards, however, in order to include many of the early studies, listing of reports where solely champion efficiencies are reported, was essential.

We note that Figure 3 in the main manuscript and Supporting Figure 24 show good continuity between the numbers of reports across the transition time (2019 to 2020) between the two search methods, suggesting that these searches cover the field sufficiently comprehensively.

Subsequently, we placed a couple of criteria on the photovoltaic devices allowed to be incorporated into the meta-analysis. First, the photo-absorber layer had to be a stoichiometric $FAPbI_3$

film, i.e. we did not include absorbers for which additives had been fully incorporated into the perovskite or treatments that would change its claimed composition away from $FAPbI_3$. For example, additives such as HI, MACl, and Cl were accepted, while others, such as MAI, MABr and CsI, were discarded. Second, any $FAPbI_3$ films of lower dimensionality were excluded from this study.

The accepted devices were then categorized into three categories: If the absorption spectra were unavailable, or if the clear determination of the presence or absence of the absorption features was deemed impossible with the data presented, then the devices associated with these spectra were categorized into "QC data unavailable". This difficulty in discerning the existence of these features can stem from the absorption spectra being too noisy, from the plotted absorption spectra not covering the energetic range where these features typically exist and are prominent (e.g. shown absorption range only covering the onset where these features are weak), or from the plots being too crowded with overlaid absorption spectra of multiple films being studied to allow for clear data extraction and analysis. However, if the absorption spectra were smooth and clear enough for an accurate evaluation, then the films, and associated photovoltaic devices, were either categorized into "QC present", or "QC absent". On numerous occasions, the films were easily categorized by eye into one of these two categories if the absorption features were clearly detectable, or if it was clearly apparent that no modulations or features existed. When this was not the case, the data were extracted from the published absorption spectra using the DigitizeIt software and the analysis of decoupling any existing features and calculating the spectra area under the peaks was carried out as described above. Accordingly, if the absorption spectrum of a $FAPbI_3$ film had a spectral area under the peaks exceeding the assigned threshold value of 1% of the full spectral area, then the features were deemed as "QC present", otherwise they were deemed as "QC absent".

We note that owing to the wide variety of reported processing methods used to fabricate $FAPbI_3$ films, we were unable to disentangle correlations between the appearance of the features and any given fabrication method or solvents used. It is practically impossible to obtain clusters of data covering identical fabrication methods because approaches vary so widely, making it

impossible to extract any statistically significant information out of such an analysis.

8.1 Additional figures displaying results of meta-analysis:

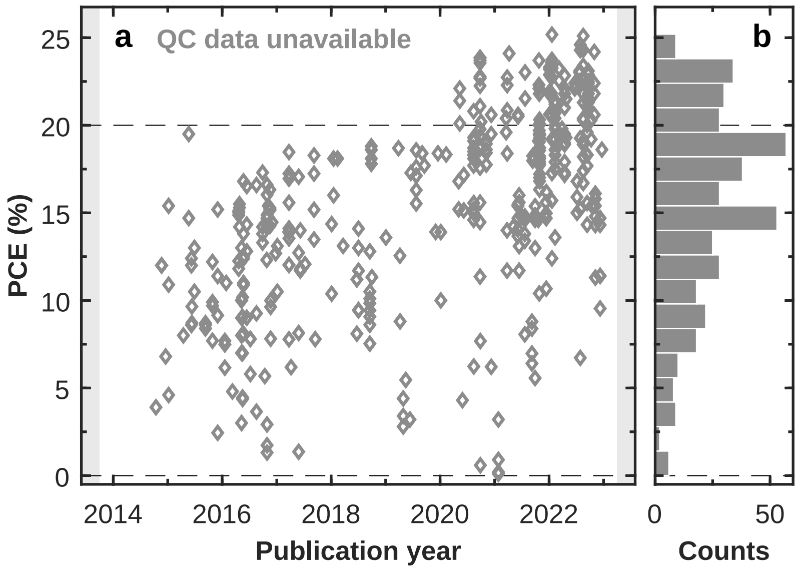

Supporting Figure 15: meta-study of device performance and absorbance features of $FAPbI_3$ thin films categorised as "QC data unavailable". a. Scatter plot of all device data. **b**. Percentage spread of the time-integrated PCE data in **a**.

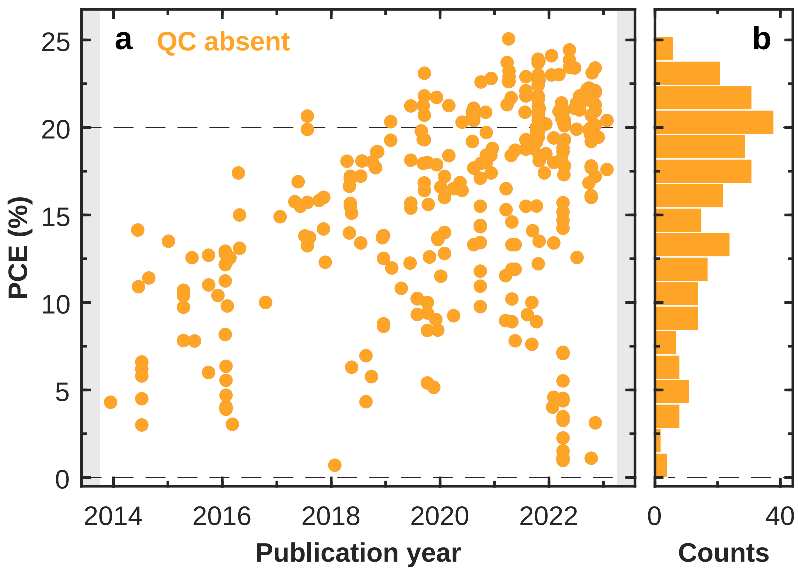

Supporting Figure 16: meta-study of device performance and absorbance features of $FAPbI_3$ thin films categorised as "QC absent". a. Scatter plot of all device data. **b**. Percentage spread of the time-integrated PCE data in **a**.

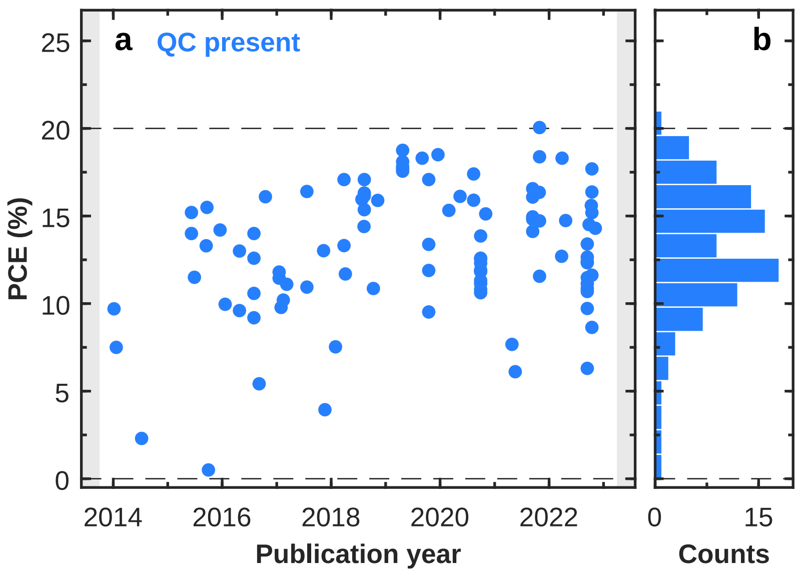

Supporting Figure 17: meta-study of device performance and absorbance features of $FAPbI_3$ thin films categorised as "QC present". a. Scatter plot of all device data. **b**. Percentage spread of the time-integrated PCE data in **a**.

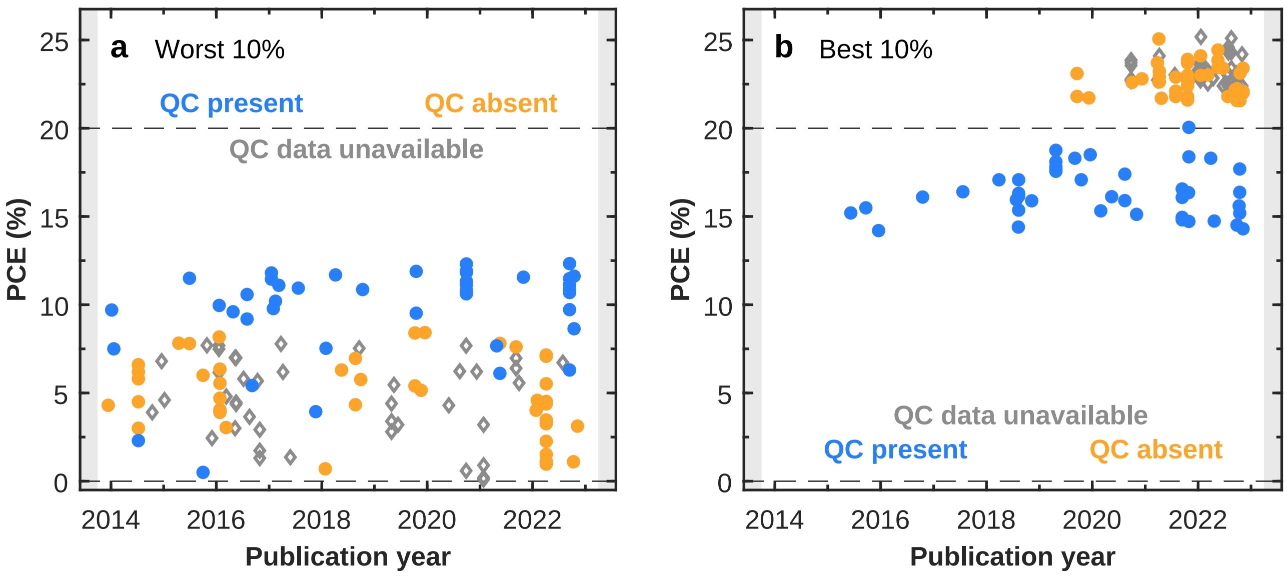

Supporting Figure 18: meta-study of device performance and absorbance features of $FAPbI_3$ thin films. Scatter plot of the devices with the lowest (**a**) and highest (**b**) 10% PCE data for each category.

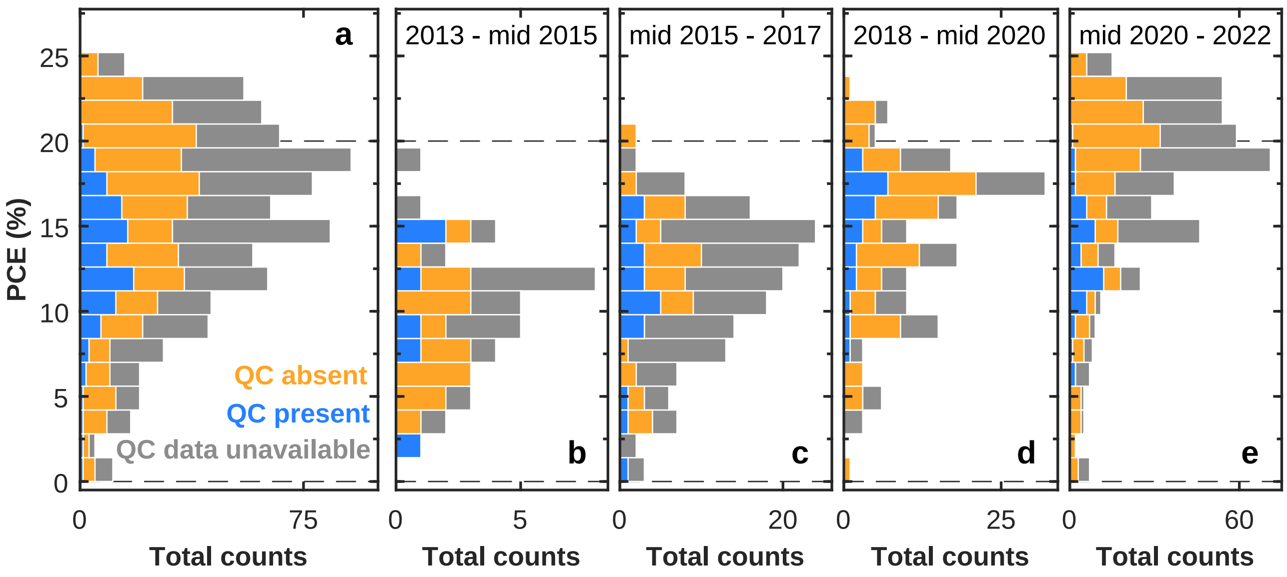

Supporting Figure 19: meta-study of device performance and absorbance features of $FAPbI_3$ thin films. a. Total counts spread of the total time-integrated PCE values presented in Main Figure 3a. Total counts spread of the 18-months time-integrated PCE values presented in Main Figure 3a from 2013 - mid 2015 (**b**), mid 2015 - 2017 (**c**), 2018 - mid 2020 (**d**), and mid 2020 - 2022 (**e**).

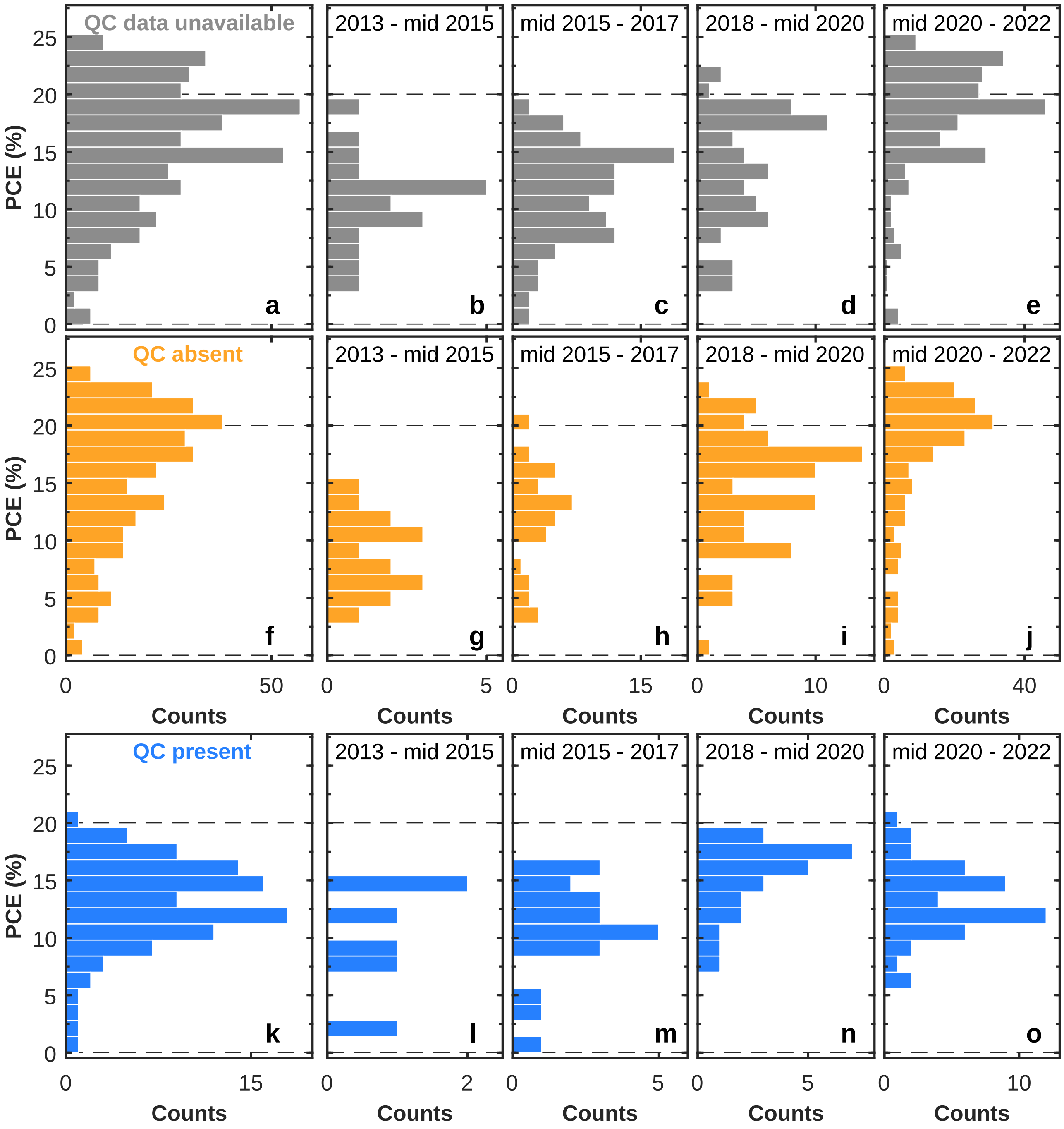

Supporting Figure 20: meta-study of device performance and absorbance features of $FAPbI_3$ thin films. Spread of the time-integrated PCE values in Main Figure 3a, for devices in the "QC data unavailable", "QC absent", and "QC present" categories.

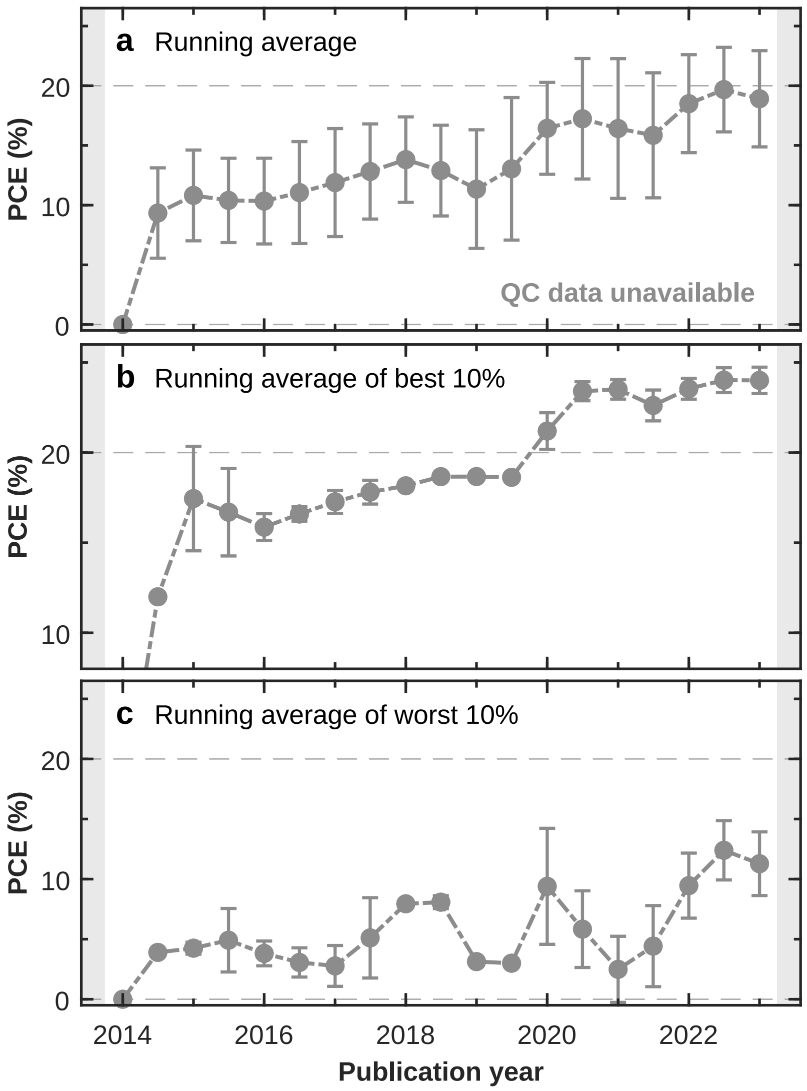

Supporting Figure 21: meta-study of device performance and absorbance features of $FAPbI_3$ thin films categorised as "QC data unavailable". Running average of total (**a**), highest 10% (**b**), and lowest 10% (**c**) PCE values for devices of each category with a one year time window. Error bars represent one standard deviation.

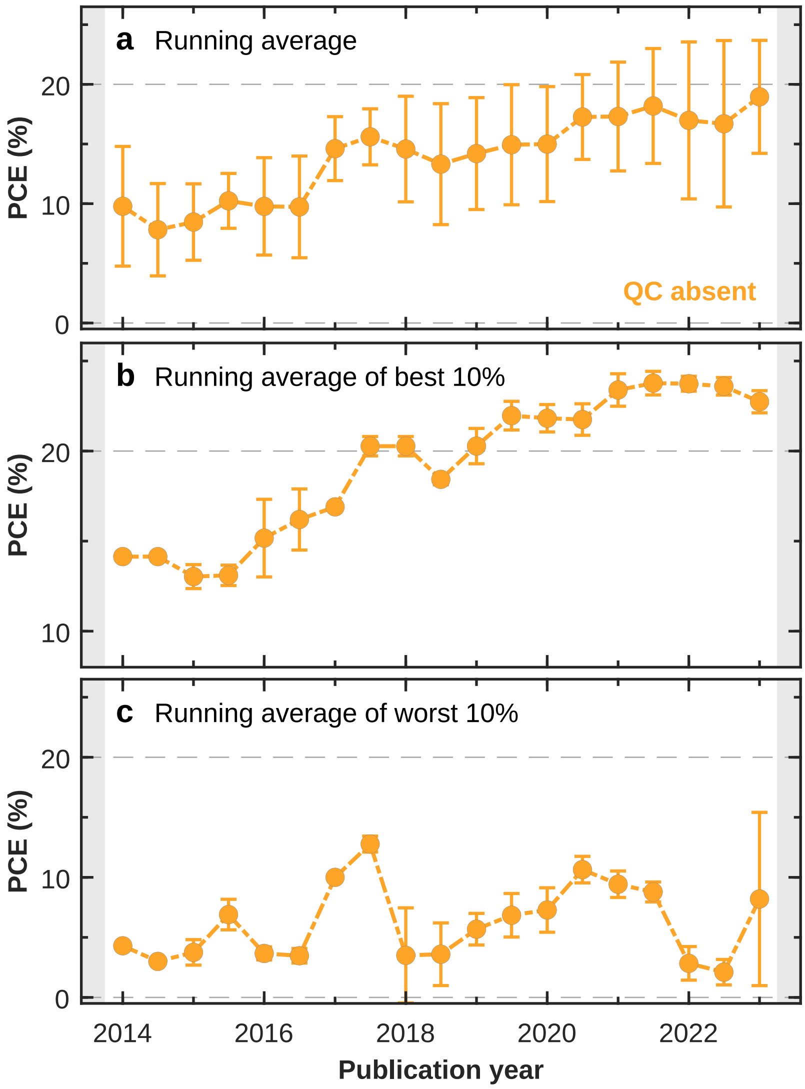

Supporting Figure 22: meta-study of device performance and absorbance features of FAPbI$_3$ thin films categorised as "QC absent". Running average of total (**a**), highest 10% (**b**), and lowest 10% (**c**) PCE values for devices of each category with a one year time window. Error bars represent one standard deviation.

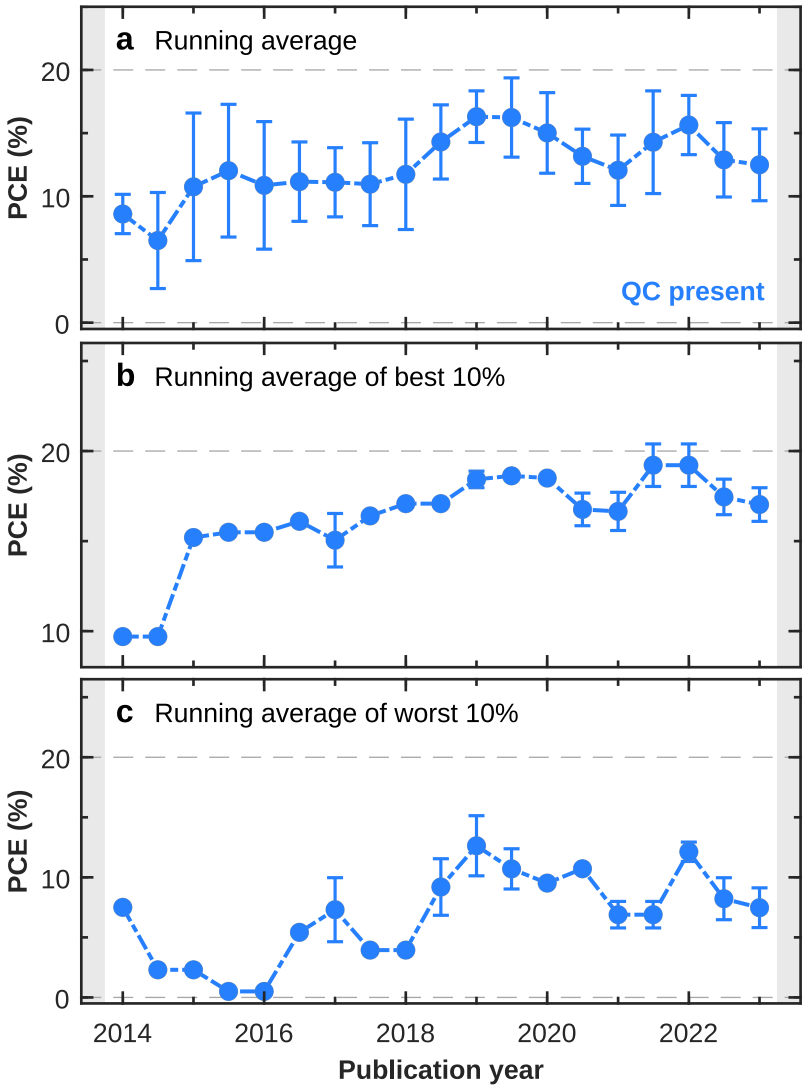

Supporting Figure 23: meta-study of device performance and absorbance features of $FAPbI_3$ thin films categorised as "QC present". Running average of total (**a**), highest 10% (**b**), and lowest 10% (**c**) PCE values for devices of each category with a one year time window. Error bars represent one standard deviation.

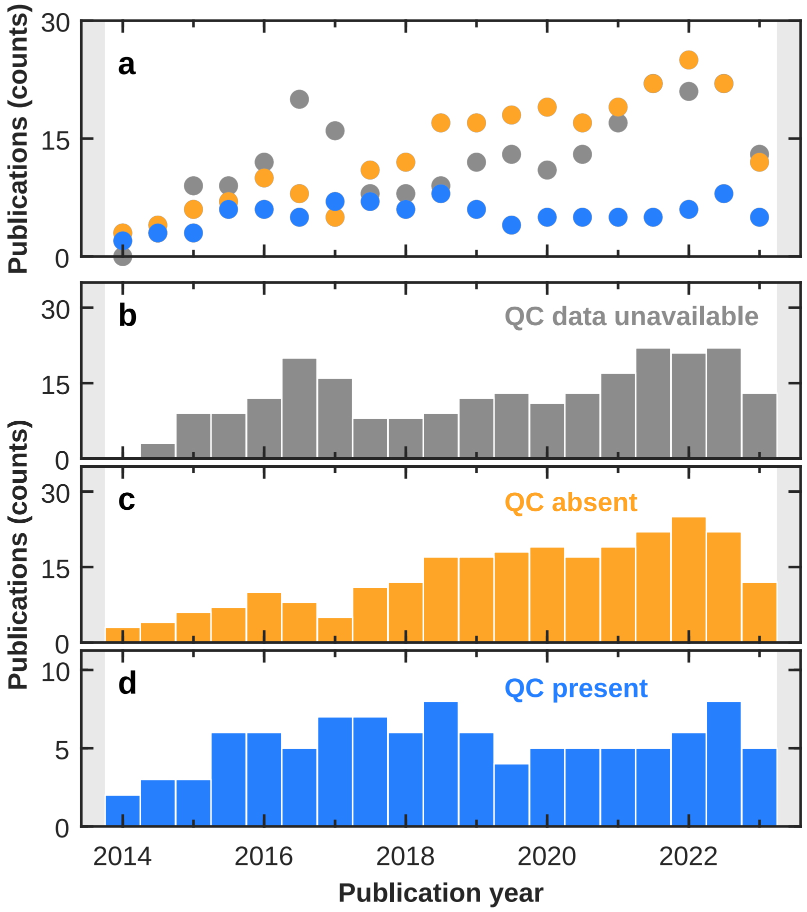

Supporting Figure 24: meta-study of device performance and absorbance features of $FAPbI_3$ thin films. a. Unique publication counts for each category with a one year time window. Unique publication counts for devices in the "QC data unavailable" (**b**), "QC absent" (**c**), and "QC present" (**d**) categories.

References

1. Kim, M. *et al.* Methylammonium chloride induces intermediate phase stabilization for efficient perovskite solar cells. *Joule* **3,** 2179–2192 (2019).

2. Jeong, J. *et al.* Pseudo-halide anion engineering for α-$FAPbI_3$ perovskite solar cells. *Nature* **592,** 381–385 (2021).

3. Kang, D.-H., Lee, S.-U. & Park, N.-G. Effect of Residual Chloride in $FAPbI_3$ Film on Photovoltaic Performance and Stability of Perovskite Solar Cell. *ACS Energy Letters* **8,** 2122–2129 (2023).

4. Min, H. *et al.* Perovskite solar cells with atomically coherent interlayers on SnO_2 electrodes. *Nature* **598,** 444–450 (2021).

5. Noel, N. K., Wenger, B., Habisreutinger, S. N. & Snaith, H. J. Utilizing nonpolar organic solvents for the deposition of metal-halide perovskite films and the realization of organic semiconductor/perovskite composite photovoltaics. *ACS Energy Letters* **7,** 1246–1254 (2022).

6. Gratia, P. *et al.* The many faces of mixed ion perovskites: unraveling and understanding the crystallization process. *ACS Energy Letters* **2,** 2686–2693 (2017).

7. Stoumpos, C. C., Malliakas, C. D. & Kanatzidis, M. G. Semiconducting tin and lead iodide perovskites with organic cations: phase transitions, high mobilities, and near-infrared photoluminescent properties. *Inorganic Chemistry* **52,** 9019–9038 (2013).

8. Trots, D. & Myagkota, S. High-temperature structural evolution of caesium and rubidium triiodoplumbates. *Journal of Physics and Chemistry of Solids* **69,** 2520–2526 (2008).

9. Cho, C. *et al.* Efficient vertical charge transport in polycrystalline halide perovskites revealed by four-dimensional tracking of charge carriers. *Nature Materials,* 1–8 (2022).

10. Elliott, R. Intensity of optical absorption by excitons. *Physical Review* **108,** 1384 (1957).

11. Wright, A. D. *et al.* Intrinsic quantum confinement in formamidinium lead triiodide perovskite. *Nature Materials* **19,** 1201–1206 (2020).

12. Elmestekawy, K. A. *et al.* Controlling Intrinsic Quantum Confinement in Formamidinium Lead Triiodide Perovskite through Cs Substitution. *ACS Nano* **16,** 9640–9650 (2022).

13. Chen, T. *et al.* Entropy-driven structural transition and kinetic trapping in formamidinium lead iodide perovskite. *Science Advances* **2,** e1601650 (2016).

14. Snaith, H. J. How should you measure your excitonic solar cells? *Energy & Environmental Science* **5,** 6513–6520 (2012).

15. Saliba, M. & Etgar, L. Current density mismatch in perovskite solar cells. *ACS Energy Letters* **5,** 2886–2888 (2020).

16. Lin, Y.-H. *et al.* A piperidinium salt stabilizes efficient metal-halide perovskite solar cells. *Science* **369,** 96–102 (2020).

17. Schutt, K. *et al.* Overcoming zinc oxide interface instability with a methylammonium-free perovskite for high-performance solar cells. *Advanced Functional Materials* **29,** 1900466 (2019).

18. Prasanna, R. *et al.* Band gap tuning via lattice contraction and octahedral tilting in perovskite materials for photovoltaics. *Journal of the American Chemical Society* **139,** 11117–11124 (2017).

19. Ghosh, D., Smith, A. R., Walker, A. B. & Islam, M. S. Mixed A-cation perovskites for solar cells: atomic-scale insights into structural distortion, hydrogen bonding, and electronic properties. *Chemistry of Materials* **30,** 5194–5204 (2018).

20. Jacobsson, T. J. *et al.* An open-access database and analysis tool for perovskite solar cells based on the FAIR data principles. *Nature Energy* **7,** 107–115 (2022).

21. *Web of Science* `https://clarivate.com/webofsciencegroup/solutions/web-of-science/`. Accessed: 2023-02-28.
